# A bacterial nutrition strategy for plant disease control

**DOI:** 10.1126/science.ady8325

**Published:** 2025-12-18

**Authors:** Shanzhi Wang, Lisong Zhu, Meng Tian, Wenyi Wu, Xu Hu, Xuan Li, Jiyang Wang, Ying Zhu, Jiaqing Xu, Baohui Mou, Jiyun Yang, Fuhao Cui, Dayong Li, Jie Cheng, Zhilong Liu, Ming-An Wang, Linlu Qi, Weiwei Jin, Zhao-Qing Luo, Pei Zhou, Yong-Hwan Lee, Brian Staskawicz, Sheng Yang He, Wenxian Sun

**Affiliations:** 1 Department of Plant Pathology, the Ministry of Agriculture Key Laboratory of Pest Monitoring and Green Management, and Joint Laboratory for International Cooperation in Crop Molecular Breeding, Ministry of Education, China Agricultural University, Beijing, China; 2 Department of Plant Pathology, College of Plant Protection, Southwest University, Chongqing, China; 3 College of Plant Protection, Jilin Agricultural University, Changchun, Jilin, China; 4 Department of Biochemistry, Duke University School of Medicine, Durham, NC 27710, USA; Department of Biology, Duke University, Durham, NC 27708, USA; 5 Department of Entomology, China Agricultural University, Beijing, China; 6 Department of Applied Chemistry, China Agricultural University, Beijing, China; 7 Department of Plant Genetics and Breeding, China Agricultural University, Beijing, China; 8 Department of Respiratory Medicine, Center of Infectious Diseases and Pathogen Biology, Key Laboratory of Organ Regeneration and Transplantation of the Ministry of Education, State Key Laboratory for Diagnosis and Treatment of Severe Zoonotic Infectious Diseases, The First Hospital of Jilin University, Changchun 130021, China; 9 Department of Biochemistry, Duke University School of Medicine, Durham, NC 27710, USA; 10 Department of Agricultural Biotechnology, Seoul National University, Seoul, Republic of Korea; 11 Department of Plant and Microbial Biology and Innovative Genomics Institute, University of California, Berkeley, Berkeley, CA, USA; 12 Department of Biology, Howard Hughes Medical Institute, Duke University, Durham, NC 27708, USA

## Abstract

*Xanthomonas* spp. cause serious diseases in over 400 plant species. The conserved AvrBs2-family effectors are among the most important virulence factors in Xanthomonads, but how AvrBs2 promotes infection remains elusive. We found that AvrBs2 is a glycerophosphodiesterase-derived synthetase that catalyzes UDP-α-D-galactose into a sugar phosphodiester, bis-(1,6)-cyclic dimeric α-D-galactose-phosphate, named xanthosan. Xanthosan is synthesized by AvrBs2 in host cells and released into apoplastic spaces. *Xanthomonas* bacteria uptake xanthosan through the XanT transporter and hydrolyze it via the XanP phosphodiesterase for nutrition. AvrBs2, XanT and XanP form a xanthosan “generation-uptake-utilization” system to provide a dedicated nutritional strategy to feed Xanthomonads. Furthermore, elucidation of the AvrBs2-XanT-XanP virulence mechanism inspired us to develop an “anti-nutrition” strategy that should be applicable to control a wide variety of *Xanthomonas* diseases.

The *Xanthomonas* genus contains various Gram-negative phytopathogenic bacteria that cause severe diseases in over 400 plant species including rice, citrus, tomato, and pepper (1, 2). *X. oryzae* pv. *oryzicola* (*Xoc*) infects rice leaves through their stomata or open wounds and causes bacterial leaf streak (BLS), one of the most severe bacterial diseases in rice (3). Although host resistance is considered an effective way to control plant diseases, very few BLS resistance gene, such as recessive *xa5*, has been reported in rice (4). Lack of effective resistance genes makes BLS a rising threat to rice production worldwide (5).

The *Xanthomonas* type III effector AvrBs2 functions as a virulence factor for many plant species and contributes to infection by *Xanthomonas* spp., including *X. phaseoli* pv. *manihotis* (previously named *Xanthomonas axonopodis* pv. *manihotis* (6, 7), *Xoc* (8, 9), and *X*. *euvesicatoria* (*Xe*. previously named *Xanthomonas campestris* pv. *vesicatoria*) (10). The *ΔavrBs2* strain is the only one out of 23 individual effector gene knockout mutants in *Xoc* that shows an obvious virulence defect in rice infection (8). Despite the importance of AvrBs2 in bacterial virulence, the exact molecular mechanism underlying AvrBs2 virulence function has remained enigmatic for decades. AvrBs2 contains a glycerophosphodiesterase (GDE)-like domain with uncertain biochemical function, and the putative GDE catalytic residues are essential for the virulence of AvrBs2 (10). However, neither the enzymatic mechanism nor the resulting enzymatic product(s) is known.

## AvrBs2 is a xanthosan synthetase

**Fig. 1.**
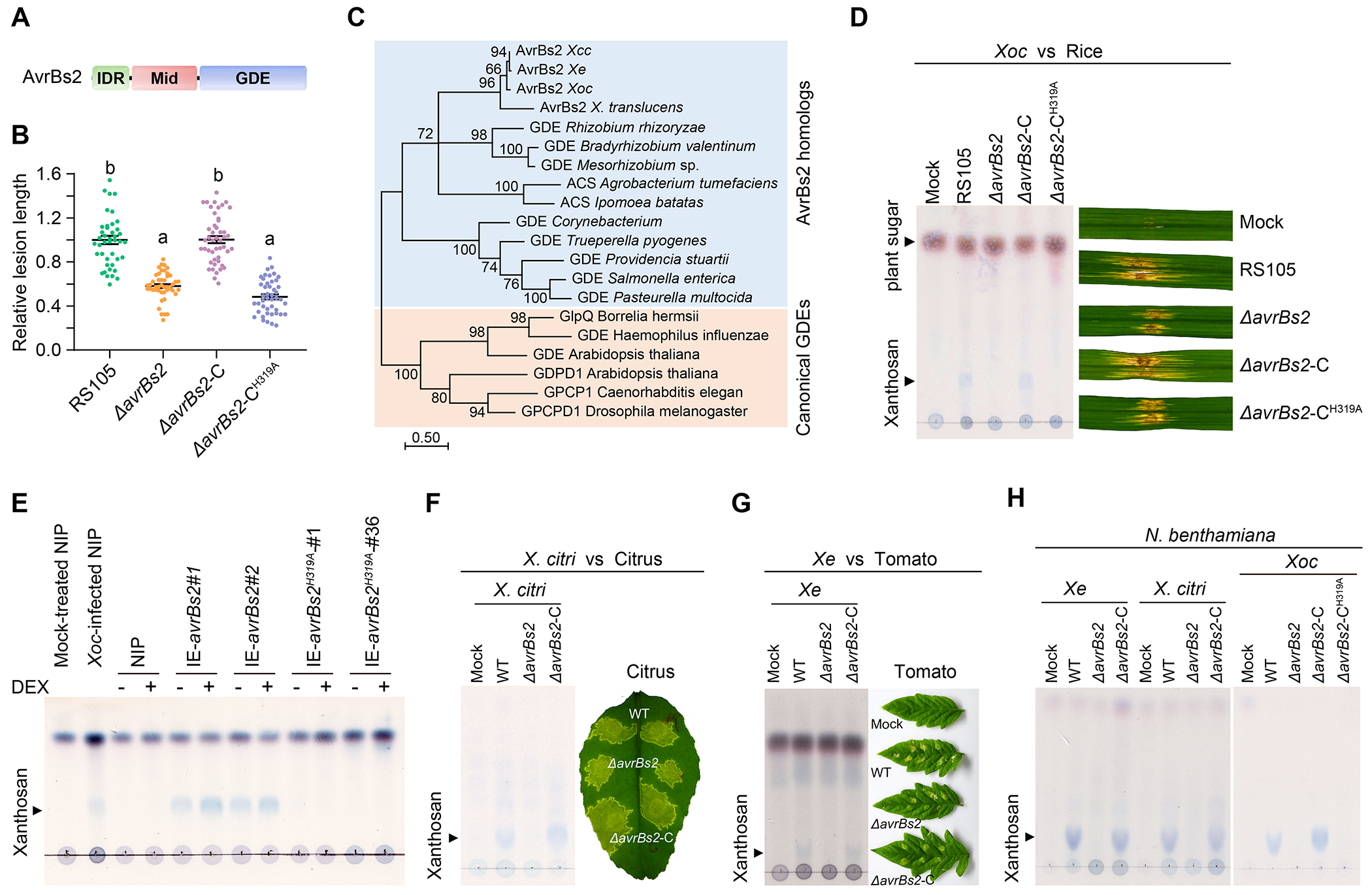
AvrBs2 homologs in Xanthomonads are involved in xanthosan biosynthesis in diverse plant species. (**A**) A schematic diagram showing that AvrBs2 contains an intrinsically disordered region (IDR), an unknown domain in the middle region (Mid), and glycerolphosphodiesterase (GDE) domain. (**B**) Disease lesion lengths on infected rice leaves by different *Xoc* strains. Data from three independent assays are normalized and shown as mean ± SE. Different letters indicate statistically significant differences (One-way ANOVA, Tukey’s honest significance test with α = 0.05). WT, wild-type strain; *ΔavrBs2*, *avrBs2* knockout strain; “-C” indicates complemented strains. (**C**) A phylogenetic tree of AvrBs2 homologs and closely related proteins. The tree was constructed by Maximum Likelihood method. The scale indicated the number of residue substitutions per site. The values indicated bootstrap values (%) from 500 replicates. *Xcc*, *Xanthomonas campestris* pv. *campestris; Xe, X*. *euvesicatoria; Xoc*, *X. oryzae* pv. *oryzicola*. (**D**) TLC analysis of xanthosan in rice leaves after *Xoc* infection. (**E**) TLC analysis of xanthosan in the wild-type, *avrBs2*- and *avrBs2*^*H319A*^-expressing rice plants. Protein expression was induced by DEX. NIP, Nipponbare. (**F**-**G**) TLC analysis to detect xanthosan in citrus leaves infiltrated with different *X. citri* strains (**F**) and in tomato leaves infected by different *Xe* strains (**G**). Right panels, disease symptoms on the infected citrus and tomato leaves. Artificially high doses of bacteria were used, which minimized AvrBs2-mediated disease symptom differences observed at low inocula as reported (10). (**H**) TLC analysis of xanthosan in *N. benthamiana* leaves at 2 days after infiltration with different *Xanthomonas* strains.

Sequence analysis showed that AvrBs2 contains a putative GDE domain at its C-terminus and is highly conserved in *Xanthomonas* (Fig. 1A and fig. S1). GDEs catalyze glycerophospholipid catabolism into glycerol 3-phosphate and alcohol (11). The putative GDE catalytic residues are essential for the virulence function of AvrBs2 in *Xe* (10). We established that AvrBs2^H319A^ with a mutation in the putative catalytic residue His319 completely abolished virulence in *Xoc* (Fig. 1B and fig. S2A). These results suggest that AvrBs2 is an active enzyme. AvrBs2 did not have detectable hydrolysis activity on glycerophosphodiester scaffold-based phospholipid in the conditions tested (fig. S2, B and C), although it may have such activity on a yet-to-be-identified substrate.

Phylogenetic analysis revealed that AvrBs2 homologs form a separate clade distinct from typical GDE subfamily proteins (Fig. 1C and fig. S1). In this clade, AvrBs2 is phylogenetically close to agrocinopine synthase (ACS), which is involved in the synthesis of agrocinopine A, a phosphodiester of sucrose and L-arabinose in *Agrobacterium* spp. (12) (Fig. 1C). Therefore, we hypothesized that AvrBs2 might have catalytic activity to synthesize certain sugar-phosphodiester compound(s). To test this hypothesis, thin layer chromatography (TLC) assays were performed to detect sugar derivatives in *Xoc-*infected rice leaves by staining with the diphenylamine-aniline-phosphoric acid reagent (13). A putative sugar compound, hereafter named “xanthosan”, was detected in the diseased leaves after infection with the wild-type *Xoc* strain, but was not detected in leaves infected with *ΔavrBs2*, *ΔavrBs2-*C^H319A^, *ΔhrcC* or *ΔhrpF* (Fig. 1D and fig. S2D).

To investigate whether AvrBs2 is involved in xanthosan biosynthesis inside plant cells, we generated transgenic rice lines expressing AvrBs2 or AvrBs2^H319A^ under the control of a dexamethasone (DEX)-inducible promoter (8) (fig. S2E). Xanthosan was detected in AvrBs2-expressing transgenic plants, but not in the AvrBs2^H319A^-expressing rice plants (Fig. 1E). A low level of xanthosan was detected in the *avrBs2* transgenic plants without DEX induction, which is likely caused by leaking basal expression of AvrBs2. Consistent with this basal expression, the *avrBs2* transgenic plants without DEX treatment were more susceptible to *ΔavrBs2* than the wild-type and *avrBs2^H319A^*-transgenic rice plants (fig. S2F). Altogether, these results indicate that AvrBs2 functions as an enzyme involved in xanthosan biosynthesis inside plant cells.

We further tested whether xanthosan was present in host plant species after infection by other economically important *Xanthomonas* pathogens. Xanthosan was detected in citrus and tomato leaves infected by *X. citri* and *Xe*, respectively, whereas no xanthosan was detected in the leaves after infection with their respective *ΔavrBs2* mutants (Fig. 1, F and G). Moreover, both *Xe* and *X*. *citri* produced xanthosan in *N. benthamiana* leaves in an *avrBs2*-dependent manner (Fig. 1H). These data indicate that AvrBs2 homologs in Xanthomonads have a conserved function in synthesizing xanthosan.

## Xanthosan is bis-(1,6)-cyclic dimeric α-D-galactose-phosphate

To identify the structure of xanthosan, a method was developed to purify xanthosan from *avrBs2-*expressing rice plants (fig. S3 A and B). We found that xanthosan was enriched in the aqueous phase after the homogenate of rice seedlings was incubated with 50% phenol (fig. S3B). Subsequently, we purified xanthosan using different concentrations of ethanol (fig. S3B). Next, ethanol-precipitated xanthosan was sequentially purified by a molecular sieve and anion-exchange chromatography (fig. S3B). Liquid chromatography showed purified xanthosan was pure enough for structure determination (fig. S3C).

Purified xanthosan was then subject to nuclear magnetic resonance (NMR) analysis. The ^13^C-NMR spectrum revealed that xanthosan contains a single type of hexopyranose because only six peaks were detected, including typical chemical shifts representing anomeric carbons (C1) at δ = 95.90 ppm, terminal carbons (C6) at δ = 63.35 ppm and C2-C5 at δ = 68.09 ~71.01 ppm (fig. S4A). The ^1^H-NMR spectrum of xanthosan exhibited typical chemical shifts of saccharides, including the anomeric proton resonance at δ = 5.4 ppm and the ring proton resonances at δ = 3.7 ~ 4.1 ppm (fig. S4B). The ^1^H-DOSY experiment confirmed that these peaks came from a single compound (fig. S4C). Next, resonances of proton and carbon atoms in xanthosan were determined based on the ^1^H-^13^C 2D-HSQC spectrum (fig. S4D). The *J*-coupling constants of protons on C3, C4 and C5 in the ^1^H-NMR spectrum of xanthosan, which are the key features of D-galactose unit (14), were consistent with those identified in α-D-galactose-1-phosphate (figure S4, B and E). The chemical shift of anomeric carbon was consistent with D-galactose in the α-configuration (15). Together, the hexose unit in xanthosan was identified as α-D-galactose. The ^31^P-NMR spectrum showed that xanthosan contained a single type of phosphate group (fig. S4F), and the ^1^H-^31^P HSQC spectrum demonstrated that the phosphate group in xanthosan forms phosphodiester bonds with C1 and C6 (fig. S4G). In addition, the molecular formula of xanthosan was determined as C_12_H_22_O_16_P_2_ by mass spectrometry (observed *m*/*z* [M-H]^−^ = 483.03050, calculated for C_12_H_21_O_16_P_2_ = 483.031031) (fig. S4H), which is consistent with a symmetrical cyclic-di-hexose-phosphate. Based on NMR and MS data, xanthosan was identified as bis-(1,6)-cyclic dimeric α-D-galactose-phosphate (fig. S4I). Furthermore, HILIC-ESI-MS assays confirmed that the identified molecule ([M-H]^−^ = 483.031) accumulated in rice leaves infected by *avrBs2*-carrying *Xoc* strains, but was not detected in rice leaves infected by the *ΔavrBs2* or *ΔavrBs2-*C^H319A^ mutant strains (fig. S5).

## AvrBs2 catalyzes UDP-α-D-galactose into xanthosan

**Fig. 2.**
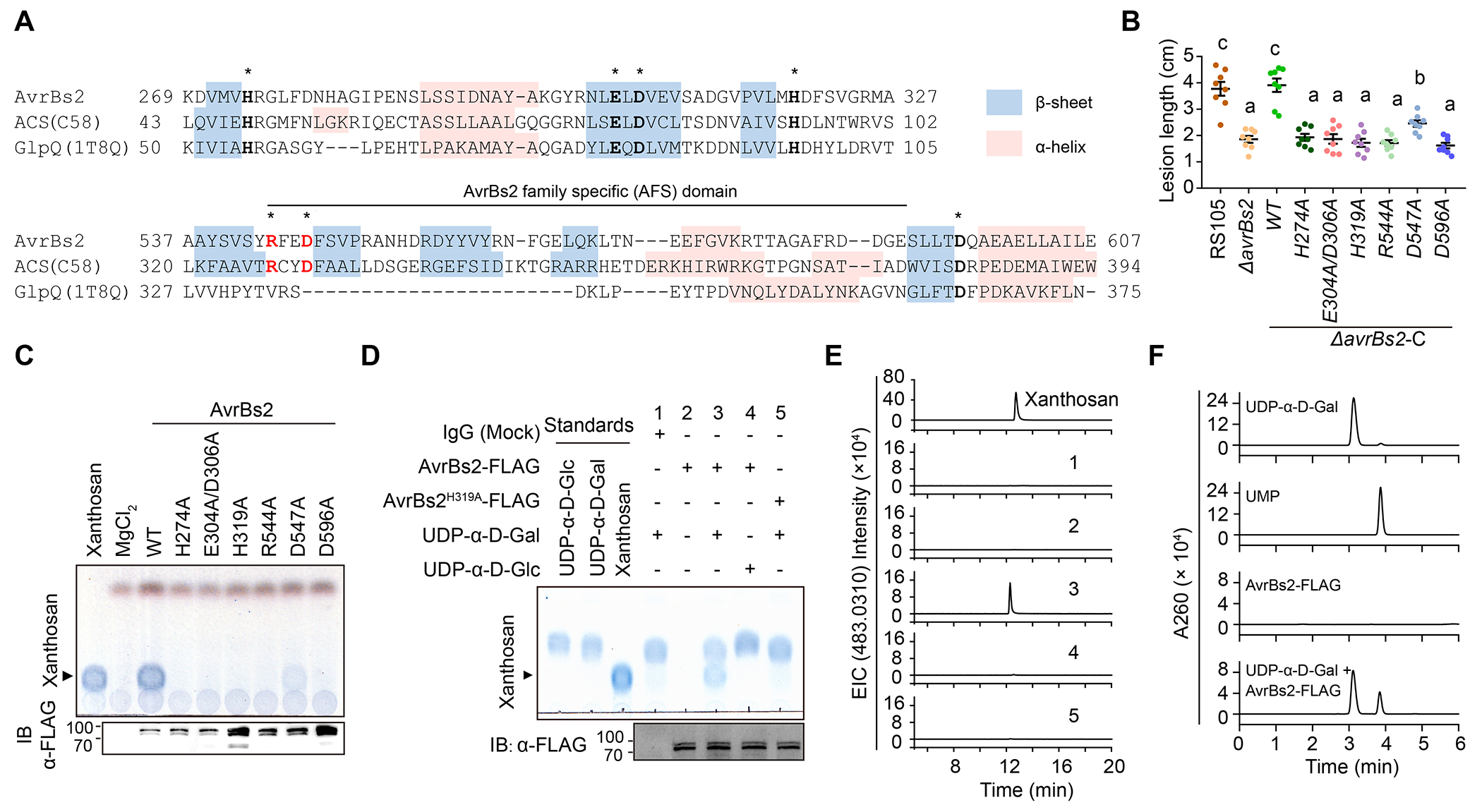
AvrBs2 catalyzes xanthosan synthesis using UDP-α-D-galactose as a substrate. (**A**) Alignment of amino-acid sequences composing the catalytic pocket in AvrBs2, ACS and a canonical GDE, GlpQ (1T8Q). “*” indicate the predicted catalytic sites. AvrBs2 family-specific motif is also indicated. (**B**) Disease lesion lengths on rice leaves after infection of the wild-type, *ΔavrBs2* and complemented strains expressing AvrBs2 or its mutants on putative catalytic sites. Data are shown as mean ± SE. Different letters indicate statistically significant differences (one-way ANOVA, Tukey’s honest significance test with α = 0.05). (**C**) TLC assay to detect xanthosan in *N*. *benthamiana* transiently expressing AvrBs2 and its catalytic mutants. (**D-E**) TLC (**D**) and HILIC-ESI-MS (**E**) assays to detect in vitro xanthosan synthesis by immunoprecipitated AvrBs2-FLAG. The numbers “1-5” indicated the same reactions in Fig. 2D and 2E. FLAG-tagged proteins were detected by immunoblotting. HILIC-ESI-MS was performed in a gradient elution procedure. (**F**) Liquid chromatograph to detect UMP as a side product in Fig. 2D.

AlphaFold prediction revealed that AvrBs2 includes a C-terminal GDE-like domain (fig. S6A). Sequence alignment showed that the AvrBs2 clade proteins, including AvrBs2 and ACS, contain an extra AvrBs2-family specific motif within the catalytic center of the GDE domain (Fig. 2A and fig. S6B). We hypothesized that the two conserved amino acid residues in the AvrBs2-family specific motif of AvrBs2, R544, and D547, may contribute to a unique catalytic mechanism along with the canonical GDE catalytic residues, including H274, E304, D306, H319, and D596 (fig. S6B). Site-directed mutagenesis revealed that R544 and canonical GDE catalytic sites were all essential for the virulence function and the catalytic activity of AvrBs2, while the AvrBs2^D547A^ mutation caused obviously reduced xanthosan synthesis activity and attenuated *Xoc* virulence (Fig. 2, B and C). These data suggest that, although AvrBs2 shares some aspects of the canonical GDE catalytic mechanism, it has unique features. Based on the acid-base catalysis mechanism of canonical GDEs (16), we speculate that AvrBs2 catalyzes xanthosan synthesis using certain α-D-galactose phosphodiesters as a substrate. After testing multiple putative substrates, we found that AvrBs2-FLAG, but not AvrBs2^H319A^-FLAG, immunoprecipitated from protein extracts of the transgenic plants was able to synthesize xanthosan using UDP-α-D-galactose, which is generated in the cytosol of plant cells and works as a nucleotide sugar donor to synthesize cell wall polysaccharides (17), as a substrate in vitro (Fig. 2, D and E).Consistent with these results, UMP was detected as a side product (Fig. 2F). By contrast, AvrBs2 failed to catalyze UDP-α-D-glucose into xanthosan (Fig. 2, D and E). Altogether, these results indicate that AvrBs2 converts UDP-α-D-galactose into xanthosan.

## Both *xanT* and *xanP* genes adjacent to *avrBs2* are crucial for *Xoc* virulence

To understand how xanthosan functions in *Xoc* infection in rice, we investigated the functionally unknown genes in the conserved *avrBs2* gene cluster in *Xanthomonas* (fig. S7A). Among them, *xanR* encodes a putative ROK (repressor, ORF and kinase) family transcriptional regulator (18); *xanT* encodes a putative TonB-dependent transporter, which is predicted to be an outer membrane transporter to absorb various nutrients, such as ferric iron, vitamin B12 and sugars (19); *xanP* encodes an unidentified 2H-phosphoesterase superfamily protein (20). RT-PCR showed that *xanT* and *xanP* are in a polycistron (fig. S7B).

**Fig. 3.**
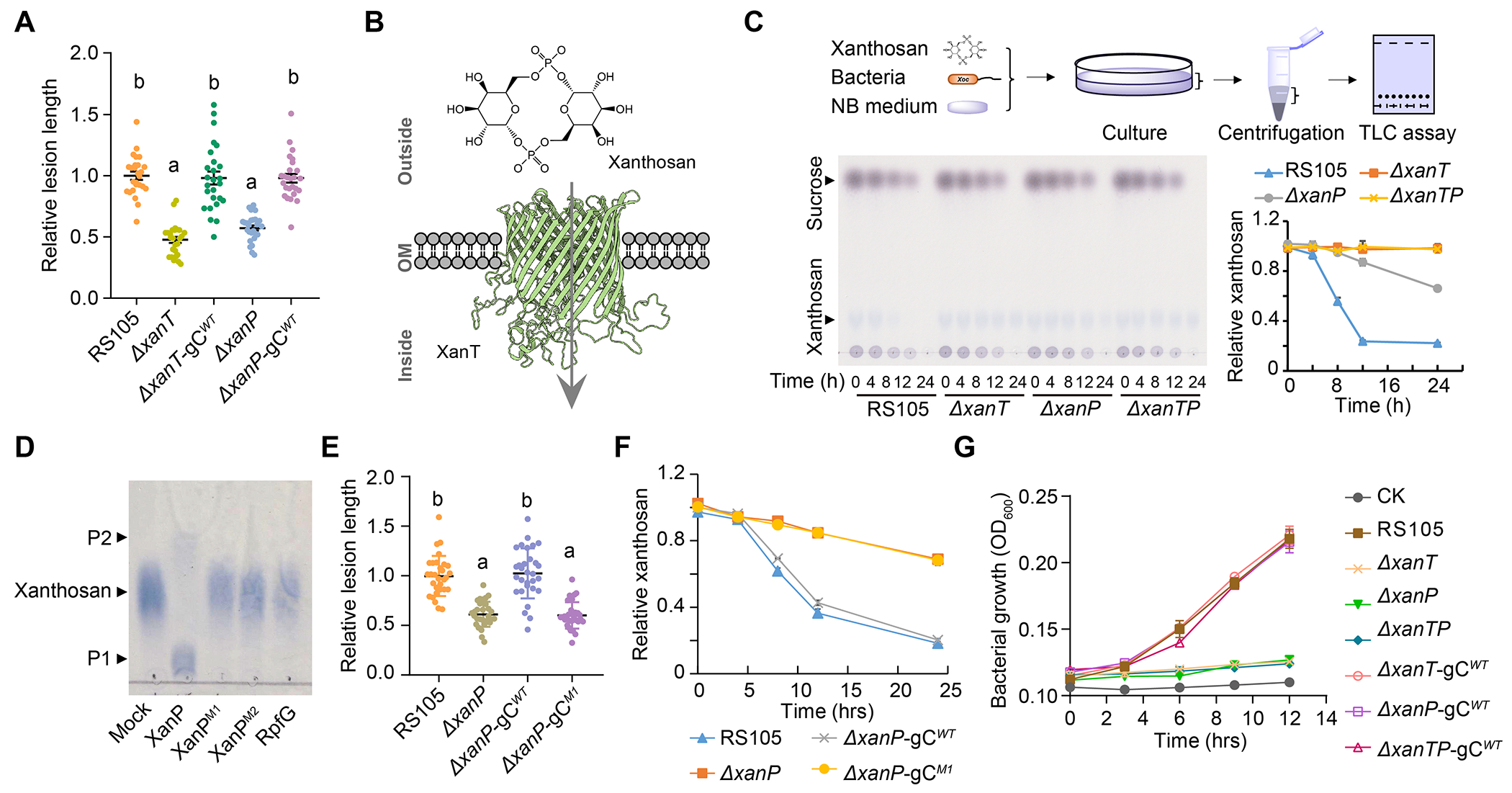
*Xoc* uptakes and hydrolyzes xanthosan as a nutrient through the XanT-XanP module. (**A**) Disease lesion lengths on rice leaves infected by different *Xoc* strains. “gC” indicates genomic complementation in situ. (**B**) XanT was predicted as an outer-membrane transporter. (**C**) Xanthosan uptake assay for different *Xoc* strains. Upper panel, a schematic diagram showing the procedure of the assay. Xanthosan in the supernatants was quantified by TLC assays (lower image and chart). (**D**) In vitro hydrolysis of xanthosan by purified His-XanP and its variants. His-XanP^M1^, His-XanP^H99A/T101A^; His-XanP^M2^, His-XanP^H204A/S206A^; P1 and P2, the hydrolytic products. His-RpfG was used as a negative control. (**E**) Disease lesion lengths on rice leaves infected by *xanP-*knockout and complemented strains. (**F**) Xanthosan uptake assay for *xanP-*knockout and complemented strains. (**G**) Growth curve assay of the indicated *Xoc* strains in the modified XVM2 minimal medium supplemented with 0.05 mM sucrose and 5 mM xanthosan. In **A** and **E**, different letters indicate statistically significant differences (One-way ANOVA, Tukey’s honest significance test with α = 0.05).

We generated *xanR-*, *xanT-* and *xanP*-knockout mutants and found that single- and double-knockout mutants of *xanT* and *xanP* genes produced shorter disease lesions and led to smaller bacterial populations in rice leaves than the wild-type and complementation strains (Fig. 3A and fig. S7, C to F). Knockout of *xanR* did not significantly alter *Xoc*-caused lesion length in inoculated rice leaves (fig. S7G), and *xanR* was absent in some *Xanthomanas* strains, such as *Xanthomonas cynarae* (fig. S7A). These data indicate that both *xanT* and *xanP* are important for *Xoc* virulence. Additionally, *ΔavrBs2* caused significantly shorter lesions and a smaller bacterial population on the inoculated leaves than single- and double-gene knockout mutants of *xanT* and *xanP*. Knockout of *xanT* and *xanP* in *ΔavrBs2* background did not cause further reduction in bacterial virulence compared to the *ΔavrBs2* mutant (fig. S7, H to J). These results indicate that *avrBs2* has an epistatic effect on *xanT* and *xanP.* During *Xoc* infection, *avrBs2* and *xanT*/*xanP*, but not *xanR*, were upregulated independently of AvrBs2 enzymatic activity (fig. S7, K to N). This unexpected finding suggests that *xanT*/*xanP* transcription is not induced by the AvrBs2-dependent metabolite xanthosan or its derivatives.

## The XanT-XanP module transports xanthosan into *Xoc* cells

We postulated that XanT might function as a transporter for xanthosan uptake into bacteria (Fig. 3B), while XanP may be involved in xanthosan use inside bacteria. Xanthosan uptake assays showed that xanthosan decreased more rapidly in the wild-type *Xoc* culture medium than that in the *ΔxanP* culture medium when the strains were cultured in xanthosan-containing medium (Fig. 3C and fig. S8, A and B). By contrast, no xanthosan reduction was observed in the medium of the *ΔxanT* or *ΔxanTP* strain (Fig. 3C and fig. S8C). However, sucrose in the culture media of these strains showed similar consumption rates, suggesting that these bacteria have equivalent growth potential (fig. S8D). In addition, xanthosan was detected in cell pellets of the *xanT*^+^ strains, but not in cell pellets of *ΔxanT* strains when these strains were incubated in xanthosan-containing medium (fig. S8E). These results suggest that XanT functions to uptake xanthosan into bacterial cells.

*Xoc* bacteria colonize extracellular (apoplastic) spaces during infection and are spatially separated from xanthosan produced intracellularly by AvrBs2. We speculated that AvrBs2-synthesized xanthosan may be released to plant apoplasts for bacterial uptake. Xanthosan was indeed detected not only in the apoplastic fluids but also in the culture medium of *avrBs2*-expressing rice seedlings (fig. S8, F and G). Moreover, xanthosan was detected in the apoplastic fluids of infected rice leaves at 48 h post infiltration (hpi) with *Xoc* and complemented (*ΔavrBs2*-C) strains, but not in leaves infected by the *ΔavrBs2* or *ΔavrBs2*-C^H319A^ mutant strain (fig. S8H). Besides, the tested bacterial strains had similar population sizes and did not cause obvious ion leakage at 48 hpi (fig. S8, I and J). These results indicate that AvrBs2-synthesized xanthosan is released from plant cells into the apoplasts. HILIC-ESI-MS assays showed that xanthosan over-accumulated (4.4- and 2.5-folds more) in the apoplastic fluids of rice leaves infected by the xanthosan uptake defective mutants *ΔxanT* and *ΔxanP*, respectively, compared with the wild-type *Xoc*-infected leaves (fig. S8, K and L). These results indicate that the XanT-XanP system is required for the import of xanthosan from the host apoplast into *Xoc* bacteria for consumption.

## XanP functions as a phosphodiesterase to hydrolyze xanthosan

XanP contains two conserved H×T/S catalytic motifs forming a putative 2H-phosphoesterase catalytic pocket (fig. S9, A and B). We examined the phosphodiesterase activity of XanP in vitro to investigate whether XanP hydrolyzes xanthosan for bacterial utilization. Purified His-XanP efficiently cleaved xanthosan to generate at least two types of hydrolytic products. Xanthosan was not degraded by His-XanP^H99A/T101A^ (His-XanP^M1^) or His-XanP^H204A/S206A^ (His-XanP^M2^), in which the two conserved catalytic residues were mutated (Fig. 3D). His-RpfG, a phosphodiesterase specifically cleaving c-di-GMP (21), did not hydrolyze xanthosan (Fig. 3D). XanP did not cleave other tested phosphoester compounds, including NAD, NADP, Gal-1-P, ATP, ADP, GTP, GDP, NBD-PC, and NBD-LysoPC, indicating that XanP is a xanthosan-specific phosphodiesterase (fig. S9, C and D).

Next, the hydrolytic products of xanthosan were isolated through anion-exchange chromatography (fig. S9E). The product with a stronger polarity and slower motility in a TLC assay was determined as C_12_H_24_O_17_P_2_ by ESI-FT ICR-MS (fig. S9F), suggesting that it is a ring-opening derivative of xanthosan, pGal-pGal, formed by incomplete hydrolysis at one phosphodiester bond. In this product, C1 and C6-linked hydrogens were identified with ^1^H-, ^13^C- and ^1^H-^13^C HSQC NMR spectroscopy (fig. S9, G–I). ^31^P- and ^1^H-^31^P HSQC NMR spectra revealed that the product was formed by cleavage at C6-linked phosphoester bond in xanthosan (fig. S9, J and K). Furthermore, the TLC assay showed that the other product migrated similarly to Gal-1-P, but not Gal-6-P, suggesting that the completely hydrolyzed product is formed by breaking two phosphodiester bonds (fig. S9L). These assays support that XanP consecutively hydrolyzes two phosphodiester bonds in xanthosan, thus forming an intermediate product pGal-pGal and the final product Gal-1-P (fig. S9M). Together with xanthosan uptake assays in Fig. 3C and fig. S8E, these results suggest that the lack of XanP-mediated degradation and use of xanthosan in bacterial cells negatively affects xanthosan uptake from the external medium.

To detect whether the hydrolytic activity of XanP is essential for *Xoc* virulence, the *ΔxanP* mutant was complemented with the full-length *xanP* gene or a catalytically deficient gene variant *xanP^M1^*. The *xanP* gene, but not *xanP^M1^*, completely restored bacterial virulence in rice and the ability to utilize xanthosan (Fig. 3, E and F and fig. S9N), indicating that the phosphodiesterase activity of XanP is crucial for bacterial virulence and xanthosan metabolism.

## *Xoc* utilizes xanthosan as a nutrient

XanP hydrolyzes xanthosan into Gal-1-P, a common sugar phosphate in prokaryotic and eukaryotic cells. Gal-1-P is utilized in bacteria through transforming into nucleotide sugars to build cell structure or hydrolyzing into D-galactose for energy (22, 23). Therefore, we speculated that *Xoc* uses xanthosan as a carbon source for growth similarly to sucrose and D-galactose (fig. S10A). Cell growth assays showed that the *ΔxanTP*, *ΔxanT* or *ΔxanP* mutant did not grew in XVM2 minimal medium containing 0.05 mM sucrose and 5 mM xanthosan, whereas the wild-type and genomic complemented strains grow well in the same medium (Fig 3G). As a control, the wild-type or *ΔxanTP Xoc* strain did not grow in the modified XVM2 medium supplemented with 0.05 mM sucrose or 5 mM xanthosan (fig. S10, B and C), whereas the tested strains grew well at similar growth rates in the XVM2 medium containing 5 mM sucrose (fig. S10D). In addition, sucrose (0.05mM) induced the expression of *xanT* and *xanP* in XVM2 medium (fig. S10, E and F), and the *Xoc* strain with constitutive expression of *xanTP* grew well in XVM2 medium supplemented with 5 mM xanthosan as sole sugar source (fig. S10G). These results indicate that *Xoc* uses xanthosan as a nutrient for growth, but requires traces of sucrose as a carbohydrate signal for the plant environment. Additionally, we found that xanthosan was not degraded at 3 days after infiltration into *N. benthamiana* leaves, indicating that plant cells are unable to metabolize xanthosan (fig. S10, H and I). These results suggest that xanthosan is a specific nutrient for *Xanthomonas* and that *Xoc* may use the xanthosan nutrition system to compete with host plants for nutrients.

## Anti-xanthosan strategy for controlling *Xanthomonas* diseases in plants

**Fig. 4.**
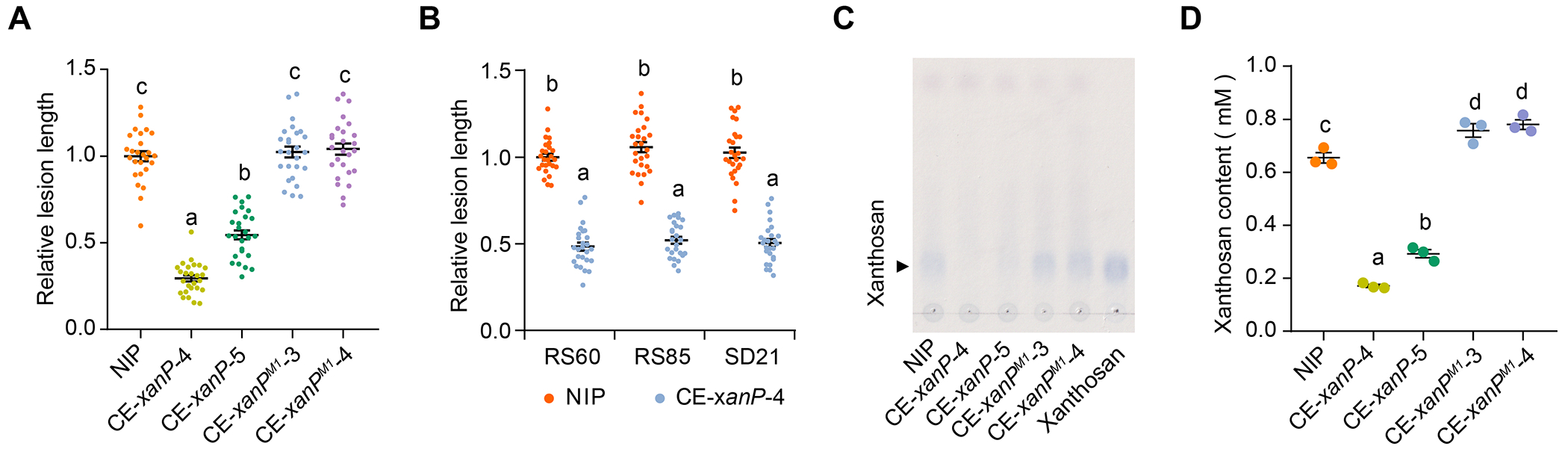
Anti-xanthosan strategy to enhance rice resistance to *Xoc* infection. (**A**-**B**) Relative lesion lengths of BLS on the wild-type, *xanP*- and *xanP*^*M1*^-expressing transgenic plants after inoculation with the *Xoc* strains RS105 (**A**) and RS60, RS80, and SD21 (**B**). Data are shown as mean ± SE. (**C**-**D**) Detection of xanthosan in the wild-type, *xanP*- and *xanP*^*M1*^-expressing transgenic plants after *Xoc* infection. TCL assay to detect xanthosan in rice leaves infected by different *Xoc* strains (**C**). Xanthosan was quantified by densitometry (n = 3) (**D**). In **A**, **B**, and **D**, different letters indicate significant difference (one-way ANOVA, Tukey’s honest significance test with α = 0.05).

The finding that XanP specifically degrades xanthosan inspired us to attempt to enhance bacterial leaf streak resistance in rice plants through transgenic expression of XanP. We generated independent XanP- and XanP^H99A/T101A^-expressing rice lines (fig. S11A). Leaf streak resistance was significantly enhanced in the *xanP* transgenic rice plants compared with the wild-type plants after inoculation with *Xoc* RS105, whereas the XanP^H99A/T101A^-expressing transgenic lines were as susceptible to *Xoc* infection as the wild-type plants (Fig. 4A and fig. S11B). In addition, XanP-expressing plants exhibited shorter disease lesions than did the wild-type plants after inoculation with other *Xoc* strains, including RS60, RS85 and SD21 (Fig. 4B and fig. S11, C to E), indicating that ectopic expression of XanP confers rice plants with non-race-specific resistance to *Xoc*. Consistent with the disease resistance phenotype, the XanP-expressing plants accumulated much less xanthosan than the wild-type and XanP^H99A/T101A^-expressing plants after *Xoc* infection (Fig. 4, C and D). Besides, the XanP-expressing plants showed no significant difference from the wild-type plants in agronomic traits including plant height, leaf width, tiller number, seed size and 100-grain weight (fig. S12, A to F), indicating that ectopic expression of XanP does not affect rice growth and development. Furthermore, we analyzed the metabolites in the wild-type and XanP-expressing transgenic rice seeds by LC-MS/MS. Among 191 and 178 annotated metabolites in all metabolites identified by LC-ESI-MS/MS in positive and negative ion models, respectively, only 1 and 3 metabolites are differentially expressed between the wild-type and transgenic rice seeds (fig. S12, G to K). The results indicate that XanP does not obviously alter metabolism in transgenic rice seeds. Altogether, based on the virulence mechanism of AvrBs2, we developed an “anti-xanthosan strategy” (fig. S13), which greatly enhances rice resistance against bacterial leaf streak without adverse effects on rice yield and metabolism.

## DISCUSSION

Although the bacterial effector AvrBs2 has been studied since 1990 (24), its biochemical function has remained enigmatic for decades. Canonical GDEs activate hydroxyl in H_2_O as nucleophile, which attacks and cleaves glycerophosphodiester substrate to form G3P and related alcohols (11). In contrast, AvrBs2 catalyzes the transformation of UDP-α-D-galactose into xanthosan and UMP (Fig 2, D to F). We hypothesize that AvrBs2 activates C6 hydroxyl in the galactose group of UDP-α-D-galactose as nucleophile to attack the phosphodiester bond in another UDP-α-D-galactose molecule, resulting in the formation of 1,6-galactose-phosphodiester bond and the release of UMP. Future biochemical and structural studies are needed to test this hypothesis and to gain further insights into the exact catalytic mechanism of AvrBs2 and related enzymes.

The nutrition strategy revealed in the study bear a conceptual resemblance to the *Agrobacterium* agrocinopine gene system. Instead of delivering an effector protein through the type III secretion system, Agrobacteria deliver the T-DNA (containing agrocinopine-synthesizing and catabolic genes) into the host plant cells through the type IV secretion system to synthesize agrocinopine, and utilize them through the membrane ABC transporters (25, 26). Although agrocinopine is structurally different from xanthosan and its biosynthesis mechanism remains unknown, it is striking that Agrobacteria and Xanthomonads have evolved convergent strategies to exploit host cells to make unique metabolites/nutrients to feed themselves. Furthermore, PSI-BLAST searches against the AvrBs2 sequence revealed the presence of AvrBs2-like proteins in a variety of bacterial genera, including some important human and animal pathogens, such as *Salmonella enterica*, *Corynebacterium*, *Pasteurella multocida*, *Providencia stuartii*, and *Trueperella pyogenes* (Fig. 1C). Our study begins to suggest that bacterium-forced host synthesis of species-tailored sugar phosphodiesters as nutrients might be a common strategy in host-microbe interactions.

In summary, this study not only unravels the biochemical function of AvrBs2 but also develops an “anti-xanthosan strategy” to enhance plant resistance against *Xanthomonas* infection. This research illustrates how fundamental understanding of an important virulence mechanism can lead to a broadly applicable solution to control important plant diseases. In particular, AvrBs2 is a key conserved virulence factor for important *Xanthomonas* pathogens. The “anti-xanthosan strategy” developed in this study is likely applicable to controlling a variety of serious bacterial diseases, ranging from bacterial spot of pepper and tomato (caused by *X*. *euvesicatoria*), cassava bacterial blight (caused by *X. phaseoli* pv. *manihotis*), citrus canker (caused by *X. citri*) to bacterial leaf streak of wheat and barley (caused by *X. translucens*).

## Supplementary Material

AvrBs2-supplementary files-accepted

## Data Availability

All data are available in the main text or the supplementary materials.
