# Supplementary material for: A bacterial nutrition strategy for plant disease control
Source: Science. Author manuscript; Available in PMC 2026 Jan 15. (PMC12807533; doi:10.1126/science.ady8325)
Supplement: AvrBs2-supplementary files-accepted [file NIHMS2133377-supplement-AvrBs2-supplementary_files-accepted.docx]

Supplementary Materials for

**A bacterial nutrition strategy for plant disease control**

Shanzhi Wang *et al*.

Corresponding author: Wenxian Sun, [wxs@cau.edu.cn](mailto:wxs@cau.edu.cn); Sheng Yang He, [shengyang.he@duke.edu](mailto:shengyang.he@duke.edu); Shanzhi Wang, 0801080926@cau.edu.cn.

**The file includes:**

Materials and methods

Figs. S1 to S13

Table S1

**Materials and methods**

**Plant materials and bacterial strains.** *Oryza sativa* ssp. *japonica* cv. Nipponbare was used as the wild type for generating transgenic plants. Rice plants were grown in the greenhouse. *Nicotiana benthamiana*, *Solanum lycopersicum* L. cv. Brandywine and *Citrus reticulata* cv. Orah plants were grown at 25°C under a 12h/12h light-dark cycle. *Arabidopsis thaliana* Col-0 was grown at 22°C under a 12h/12h light-dark cycle. *Escherichia coli* and *Agrobacterium tumefaciens* strains were cultured in LB medium (1% tryptone, 0.5% yeast extract, 1% NaCl) at 37°C and 28°C, respectively. *Xanthomonas* spp. were cultured in NB medium (0.5% tryptone, 0.1% yeast extract, 0.3% beef extract, and 1% sucrose) at 28°C. Antibiotics were used at the following concentrations unless specifically noted, 25 μg mL^-1^ rifampicin, 50 μg mL^-1^ ampicillin and 50 μg mL^-1^ kanamycin.

**Construction of gene-knockout and complemented strains of *Xanthomonas* spp.**

The marker-free gene deletion mutants, including *ΔavrBs2^X.citri^*, *ΔavrBs2^Xe^*, *ΔhrpF*, *ΔxanR*, *ΔxanP*, *ΔxanT^FL^* and *ΔxanTP* (*xanT* and *xanP* double-gene knockout) were constructed through homologous recombination as previously described (*8*). Briefly, upstream and downstream fragments (~1 kb) of targeting genes amplified from *Xanthomonas* genomic DNA were fused by overlap extension (SOEing) PCR. The resultant PCR products were subcloned into pUFR80 (or pK18mobsacB for *ΔhrpF*) that carries *sacB* suicide gene as a counter-selective marker (*27*). The constructed plasmids were conjugated into *Xanthomonas* strains by triparental mating. The conjugants were screened on NA plates with kanamycin. After culturing in NB medium lacking sucrose overnight, the conjugants were cultured on 5% sucrose-containing NA plates. The gene-knockout mutants were then identified from sucrose-insensitive colonies by PCR. Multiple-gene knockout strains including *ΔavrBs2ΔxanP, ΔavrBs2ΔxanT* and *ΔavrBs2ΔxanTP* were constructed in the *ΔavrBs2* background using the same procedure as described above.

Because *xanT* is located upstream of *xanT*-*xanP* polycistron, we constructed the *ΔxanT* mutant with a frameshift mutation caused by single base-pair deletion to avoid interfering with expression of *xanP*. The *xanT^ΔC606^* fragment of full-length *xanT* gene including about 1-kb upstream and downstream sequences with single 606^th^ base-pair deletion was generated by SOEing PCR and was then sub-cloned into the suicide plasmid pUFR80. The construct was conjugated into the *xanT* gene-deletion strain, *ΔxanT^FL^*, by triparental mating. The *xanT^ΔC606^* mutants were identified through PCR and sequencing, and were used for all of related assays including bacterial infection, xanthosan uptake, in planta xanthosan quantification.

The *avrBs2* homologous genes with native promoters were amplified by PCR from different *Xanthomonas* spp.. The point mutations of *avrBs2* gene were generated by site-directed mutagenesis or SOEing PCR with the primers listed in Table S1. These gene fragments were sub-cloned into the broad host-range vector pVSP61 (*28*). The *ΔavrBs2* knockout mutants were complemented with pVSP61-borne *avrBs2* genes fused to HA-coding sequence. To express *xanT* and *xanP* constitutively, the *pilA* promoter (782bp) was fused with *xanTP* fragment by SOEing PCR. The fusion product was ligated into linearized pBBRMCS2 plasmid using in-fusion method after digestion with *Age* I and *Nsi* I. These constructs were conjugated into the gene-deletion mutants by triparental mating, and the transformants were screened on kanamycin-containing NA medium plates.

The *ΔxanT*, *ΔxanP* and *ΔxanTP* mutants were genomically complemented through homologous recombination using the same procedure as construction of gene-deletion mutants. Briefly, the *xanT*, *xanP* and *xanTP* full-length genes containing about 1-kb upstream and downstream sequences were amplified by PCR. The *xanP^M1^* (*xanP^H99A/T101A^*) mutant fragment was generated by SOEing PCR. The amplified fragments were sub-cloned into pUFR80. The constructs were conjugated into the respective mutant strains by triparental mating. The constructed gene fragments were inserted into the genome through homologous recombination to generate *ΔxanT-gC^WT^*, *ΔxanP-gC^WT^* and *ΔxanTP-gC^WT^*. The primers used in this study are listed in Table S1.

**Pathogen inoculation assays**

Virulence of *Xoc* strains to rice was determined by pressure inoculation (*29*). Briefly, overnight-cultured *Xoc* strains were collected and re-suspended with 10 mM MgCl_2_ to a cell density of OD_600_ = 0.3. Cell suspensions were pressure-inoculated into rice leaves using needleless syringes. The lesion lengths were measured at ~2 weeks after *Xoc* inoculation. To evaluate bacterial population, rice leaf fragments including disease lesions (4 cm-length) were collected at the indicated time-points, and were then sterilized with 75% ethanol for 30 s. Leaf fragments were ground in 1 ml of sterile MgCl_2_ solution (10 mM), and was plated on NA plates after serial dilution. Colony forming units per disease lesion were counted after 2-d culturing.

**Protein expression and purification in *E. coli***

The open reading frames (ORFs) of *avrBs2* and *xanP* were amplified using the primers in Table S1. *avrBs2* was subcloned into pGEX-4T-3 and pCold-SUMO, while *xanP* was subcloned into pET28a. The vectors to express the AvrBs2^H319A^, XanP^M1^ (XanP^H99A/T101A^) and XanP^M2^ (XanP^H204A/S206A^) mutants were generated via site-directed mutagenesis. All constructs confirmed by sequencing were transformed into *E. coli* BL21(DE3) for protein expression.

For in vitro protein purification, 100 µM of IPTG was added into cell cultures to induce protein expression at cell density of OD_600_ = 0.6. The cells were further cultured at 16°C with shaking at 150 rpm overnight and were then collected by centrifugation at 1, 500 g for 10 min. Cell cultures were sonicated in the lysis buffer (50 mM Tris-Cl, pH 8.0, 150 mM NaCl and 10 mM imidazole) for His-tagged proteins or in the binding buffer (50 mM Tris-Cl, pH 8.0, 150 mM NaCl) for GST-tagged proteins. Cell debris was spun down by centrifugation at 13, 400 g for 10 min at 4°C, and the supernatants were loaded onto Ni-NTA His·Bind^®^ and GST·Bind^TM^ resins (Navogen, EMD Millipore, Billerica, MA), respectively. For His-tagged proteins, the resin was rinsed twice with lysis buffer and twice with washing buffer (50 mM Tris-Cl, pH 8.0, 150 mM NaCl and 20 mM imidazole). The proteins bound to the resin were eluted with elution buffer (50 mM Tris-Cl, pH 7.5, 150 mM NaCl) supplemented with 100 mM or 150 mM imidazole. For GST-tagged proteins, the resin was washed four times with binding buffer. The proteins were then eluted with elution buffer supplemented with 10 mM glutathione. For xanthosan hydrolysis assay, purified His-XanP was dialyzed against ddH_2_O at 4°C for 24 h. Purified proteins were quantified by Bradford method (*30*).

**Phospholipid hydrolysis assay**

Fluorescent phospholipid hydrolysis and thin layer chromatography (TLC) assays were performed as described previously (*31*). Briefly, 1 μg of purified His-AvrBs2 was incubated with 5 μg of fluorescent lipids (Avanti Polar Lipids, America) at 28°C for 1 h in 20 μL reaction solution (40 mM Tris-HCl, pH 7.5, 5 mM MgCl_2_, 5 mM CaCl_2_ and 0.025% Triton X-100). The sample was dried in a Speed-Vac for 30 min at 45°C, and was then dissolved in 8 μl of methanol/isopropanol/acetic acid (v/v/v, 5 : 5 : 2). The products (1 μl) were loaded on TLC silica gel 60 plates (Merck Millipore, Darmstadt, Germany), which were developed using chloroform/methanol/acetone/acetic acid/water (v/v/v/v/v, 70 : 50 : 20 : 20 : 20) for 20 min. The fluorescence was excited by UV light, and was then captured with an Azure C600 Gel Imaging System (Azure Biosystems, America).

For phospholipid hydrolysis of egg yolk, the egg yolk plate was prepared as reported with minor modifications (*32*). Briefly, fresh egg yolk (20 ml) was mixed with 60 ml of 0.85% NaCl and was then centrifuged at 2000 rpm for 2 min. The supernatant was collected as solution A. Solution B was prepared by dissolving 0.6 g agarose in 100 ml of 50 mM Tris-HCl (pH 7.5) and kept in 50℃ water bath. The solutions A, B, and 10 mM CaCl_2_ (v/v/v, 1 : 98 : 1) were mixed, and 14 ml of mixture was then poured into a petri dish. After solidification, 3-mm wells were punched in the plate. Purified proteins (50 μg) and commercial PLA2 (2 μg) as a positive control were loaded into the wells separately, and were further incubated at 28℃ for 5 h. The diameter of transparent area around the wells formed after the degradation of egg yolk was measured.

**Detection of xanthosan by thin layer chromatography**

Six-week-old rice leaves were infiltrated with *Xoc* cell suspension (OD_600_ = 0.3), and diseased lesion areas were collected at 14 days post infiltration (dpi). Four-week-old *N. benthamiana* leaves were infiltrated with suspensions of *Xanthomonas* spp. (OD_600_ = 0.1), and infiltrated leaf areas were collected at 2 dpi. Four-week-old tomato and citrus leaves were infiltrated with *Xe* and *X*. *citri* suspensions (OD_600_ = 0.1 and 0.3, respectively), and diseased lesion areas were collected at 2 and 4 dpi. To extract xanthosan, collected samples were ground into powder using liquid nitrogen. The powder was incubated with 2 volumes of phenol and ddH_2_O mixture (v/v, 1 : 1) at 65°C for 5 min after mixing with vortex. After centrifugation at 13, 400 g for 10 min at 4°C, xanthosan in the aqueous phase was purified by precipitation using absolute ethanol. The precipitants were collected by centrifugation and were then dissolved with 20 μl of ddH_2_O for TLC detection.

The DEX-inducible *avrBs2*- or *avrBs2^H319A^*-expressing transgenic rice seedlings (7-day-old) were cultured for 4 days in 1/2 MS liquid medium containing 30 μM DEX or ethanol (0.1%) as mock control. The *avrBs2-FLAG* fragment and its variants were amplified with the primers listed in Table S1 and subcloned into pGD-35S or pGD-35S-GFP vectors, which were transformed into *Agrobacterium* GV3101 competent cells. Four-week-old *N. benthamiana* leaves were agro-infiltrated with suspensions of the transformed cells (OD_600_ = 0.5). Leaf samples were collected at 2 dpi. Xanthosan was extracted from rice seedlings and *N. benthamiana* leaves as described above and was then detected by TLC assay. Xanthosan secreted into the medium was directly detected by TLC assay.

The xanthosan sample (0.8 μl) was loaded onto TLC silica gel 60 plates, which were developed in ddH_2_O/acetic acid/isopropanol (v/v/v, 1 : 1 : 3) for 25 min. The plates were soaked in the diphenylamine-aniline-phosphoric acid reagent (1 g diphenylamine, 1 ml aniline, 5 ml phosphoric acid and 45 ml acetone), and were then heated at 100°C for 10 min to show xanthosan and saccharides.

**Extraction and purification of xanthosan**

For large-scale extraction of xanthosan, DEX-inducible *avrBs2*-expressing rice seedlings were grown in 1/2 MS liquid medium supplemented with 30 μM DEX for 2 weeks, and were then ground into powder. The powder (200 g) was mixed with 400 ml ddH_2_O and 400 ml phenol by stirring and was then heated at 65°C for 30 min. After centrifugation at 10,000g for 10 min at 25°C, the aqueous phase was extracted with diethyl ether, and was then concentrated by rotary evaporation at 65°C to a final volume of 20 ml. After addition of ethanol (40 ml), the solution was centrifuged at 10,000g for 10 min at 25°C. The supernatant was collected and mixed with 120 ml ethanol to precipitate xanthosan, which was collected after centrifugation at 10,000g for 10 min at 25°C. The pellet was dissolved in 5 ml of ddH_2_O and separated on a Sephadex G-25 molecular sieve column (2.5 cm diameter × 73 cm height) using 0.01 M NH_4_HCO_3_ as mobile phase. Aliquots (10 ml) were collected consecutively. After being detected by TLC assay, xanthosan-containing aliquots were concentrated to a final volume of 5 ml by rotary evaporation at 65°C. Xanthosan was purified twice via molecular sieve columns. Next, the concentrated fraction was loaded onto a 1-ml Resource Q anion-exchange chromatography column (GE, Healthcare) pre-equilibrated with deionized water. Bound compounds were eluted in a linear gradient of 30-column volumes from ddH_2_O to an aqueous solution of 1 M NH_4_HCO_3_, and aliquots (1 ml) were collected consecutively. Xanthosan-containing aliquots were subject to three rounds of anion-exchange chromatography and were finally evaporated to powder by rotary evaporation at 65°C.

**Preparation of hydrolysate of xanthosan**

Purified xanthosan (50 mg) was incubated with 2 mg of dialyzed His-XanP at 28°C overnight. The sample was filtered through a 3 kDa-cutoff ultrafiltration tube (Millipore) by centrifugation at 4,000 rpm. The filtrate was loaded onto Resource Q anion-exchange chromatography column to separate the hydrolysates of xanthosan as described above. The fractions were detected by TLC assay, and two hydrolysate products P1 and P2 were collected separately.

To prepare the saccharide moiety of xanthosan, xanthosan (50 mg) was incubated with 4 mg of dialyzed His-XanP and 400 U of commercial calf intestine alkaline phosphatase (CIAP) (Takara, Dalian, China) overnight. The sample was filtered through a 3kDa-cutoff tube. The filtrate was loaded onto a 300-400 mesh silica gel column (2.5 cm diameter × 12.5 cm height), and eluted with ddH_2_O/acetic acid/isopropanol (v/v/v, 1 : 1 : 3).

**ESI-FT-ICR-MS**

Purified xanthosan and its hydrolysate P1 were dissolved in ultrapure H_2_O to a final concentration of 1 mg/ml. Mass spectrometry (MS) was performed using a SolariX 9.4T FT-ICR MS (Bruker Daltonics, Bremen, Germany) equipped with ESI in negative ion mode with a scan range from 50 to 1000 *m*/*z* at Institute of Chemistry, Chinese Academy of Sciences (Beijing, China).

**Nuclear magnetic resonance (NMR) spectroscopy**

Commercially purchased α-D-galactose-1-phosphate (MedChemExpress, HY-113143), purified xanthosan and its hydrolysate P1 were dissolved in D_2_O. 1D ^1^H-NMR spectrum for α-D-galactose-1-phosphate was recorded with a pulse sequence, zg30 using a 500 MHz Bruker Avance III HD NMR spectrometer at College of Science, China Agricultural University (Beijing, China), and the parameters were set as: spectral width, 20 ppm; collected data points, 65536; acquisition time, 3.28 s; recycle delay, 1 s and the number of scans, 16. NMR assays for xanthosan, including ^1^H-, ^13^C-, ^31^P-, ^1^H-DOSY and ^1^H-^13^C-HSQC-NMR spectra, were performed using a 400 MHz Bruker Avance III HD NMR spectrometer at Center of Pharmaceutical Technology, Tsinghua University, and the parameters were listed as follows. 1D ^1^H-NMR spectrum of xanthosan was recorded using a pulse sequence, zg30 with parameters: spectral width, 20 ppm; collected data points, 65536; acquisition time, 4 s; recycle delay, 1 s; and the number of scans, 16. 1D ^13^C-NMR spectrum of xanthosan was detected using a pulse sequence, zgpg30 with parameters: spectral width, 240 ppm; collected data points, 65536; acquisition time, 1.36s; recycle delay, 2 s; and the number of scans, 1024. 1D ^31^P-NMR spectrum of xanthosan was recorded using a pulse sequence, zgpg30 with parameters: spectral width, 395 ppm; collected data points, 65536; acquisition time, 0.5s; recycle delay, 2 s; and the number of scans, 16. 2D DOSY spectrum of xanthosan was recorded with a pulse sequence, ledbpgp2s with parameters: diffusion time, d20 = 200 ms; gradient lengths, p30 = 0.9 ms, p19 = 0.6 ms; eddy current delay, d21 = 5 ms; gradient ratios, gpz6 100%, gpz7 -17.13% and gpz8 -13.17%; the number of gradient steps, 32. 2D ^1^H-^13^C HSQC spectrum of xanthosan was recorded with a standard pulse sequence, hsqcetgpsi2. The HSQC parameters were set as: recycle delay, 1.5 s; acquisition time, 0.128 s; spectral widths, 10 ppm and 165 ppm with 1024 × 256 data points for ^1^H and ^13^C spectra, respectively. 2D ^1^H-^31^P HSQC NMR spectra of xanthsosan and its hydrolysate pGalpGal were recorded with a standard pulse sequence, hsqcetgpsi, using a 500 MHz Bruker Avance III HD NMR spectrometer at Shanghai Institute of Materia Medica, Chinese Academy of Sciences (Shanghai, China). The parameters were set as: recycle delay, 2.5 s; acquisition time, 0.2048 s; spectral widths, 10 ppm and 99 ppm with 1024 × 512 data points for ^1^H and ^31^P spectra, respectively.

1D ^13^C-NMR spectrum of the hydrolysate pGalpGal was recorded with a standard pulse sequence, zgdc, using a 500 MHz Bruker Avance III HD NMR spectrometer at Analytical and Testing Center, Beijing Normal University. The parameters were set as: spectral width, 237 ppm; acquisition time, 1.1 s; recycle delay, 2.0 s; and the number of scans, 1024. 1D ^1^H-, ^31^P-, and ^1^H-^31^P-HSQC NMR spectra of pGalpGal were tested with a 500 MHz Bruker Avance III HD NMR spectrometer at Shanghai Institute of Materia Medica, Chinese Academy of Sciences (Shanghai, China). 1D ^1^H-NMR spectrum of pGalpGal was recorded with a standard pulse sequence, zg30, and the parameters were set as: spectral width, 20 ppm; the acquisition time, 3.3 s; recycle delay, 1.0 s; and the number of scans, 16. 1D ^31^P-NMR spectrum of pGalpGal was recorded with a standard pulse sequence, zgpg, with the parameters: spectral width, 587 ppm; acquisition time, 0.55 s; recycle delay, 2.5 s; and the number of scans, 64. 2D ^1^H-^13^C HSQC spectrum of the hydrolysate pGalpGal was recorded with a pulse sequence, hsqcedetgpsisp2.3 using a 500 MHz Bruker Avance III HD NMR spectrometer at College of Science, China Agricultural University (Beijing, China). The HSQC parameters were set as: recycle delay, 1.5 s; acquisition time, 0.1024 s; spectral widths, 20 ppm and 220 ppm with 1024 × 256 data points for ^1^H and ^13^C spectra, respectively. Reference for chemical shifts of NMR spectra: ^1^H-NMR spectra with DHO at δ 4.70 ppm, ^13^C-NMR spectra with CD3OD at δ 49.50 ppm and ^31^P-NMR spectra with 85% phosphoric acid at δ 0.00 ppm. All NMR data were processed and analyzed using MestReNova software (Mestrelab Research S.L.).

**Isolation of apoplastic fluids from rice leaves**

Apoplastic fluids in rice leaves were isolated with an infiltration-centrifugation method with minor modifications (*33*). One-month-old rice leaves were cut into 5cm-length segments and immersed in 0.01% Silwet L77 for 2 min. After washing with deionized water for three times, leaf segments were immersed in 30 mL of 1 × PBS buffer in a 60-mL polyethylene syringe, and were manually infiltrated for 3 min. The syringe with PBS buffer and leaf segments was set in a sealant injector, and was pressured to a maximum pressurization for 3 min to infiltrate leaf segments completely. After infiltration, the leaf segments were washed with deionized water for twice and inserted into 1ml pipet tips. The tips were placed in 15 ml centrifuge tubes, and centrifuged at 6000 × g for 10 min at 4℃. The apoplastic fluid in the centrifuge tubes was collected, and was used for subsequent assays immediately or was stored in a -80 ℃ freezer.

**HILIC-ESI-MS assay to detect xanthosan in planta**

To detect xanthosan in plant cells, the *Xoc*-infected leaves were ground with liquid nitrogen. After the powder was incubated with 50% phenol, xanthosan in aqueous phase was precipitated with ethanol as described above. The pellets were dissolved in H_2_O followed by centrifugation at 12,000 g for 10 min. The supernatant was collected for HILIC-ESI-MS assay. To detect xanthosan in the apoplastic space, apoplastic fluids were directly analyzed after dilution. LC-MS was performed on an Agilent Technologies 1260 infinity LC system, coupled to an Agilent 6520 QTOF mass spectrometer (Agilent Co. Ltd., Santa Clara, CA). The samples were separated on a Poroshell 120 HILIC-Z column (2.7 μm, 2.1 mm × 100 mm, Agilent Technologies). Mobile phase A was 0.1% (v/v) formic acid in water, and mobile phase B was 0.1% (v/v) formic acid in acetonitrile. The flow rate was set as 0.3 ml/min. For detection of xanthosan in plant cell extracts and apoplastic fluids, the elution was under an isocratic condition with 80% A for 10 min. For detection of in vitro synthesized xanthosan, the elution procedure was set as 50% - 90% A for 10 min and 90% A for 20 min. MS assay was operated using negative ion ESI with scan range from *m/z* 400 to 550.

**Liquid chromatography to detect xanthosan, nucleotides and nucleotide sugars**

The purity of isolated xanthosan was determined by a LC method on a 1260 Infinity II system (Agilent). The sample (10μL) was separated on an Aminex® HPX-87H column (Bio-Rad, 9 μm, 7.8 mm × 300 mm). The flow rate was set as 0.5 ml/min. The elution buffer was 50 mM H_2_SO_4_ in water. Xanthosan was detected with a G7162A refractive index detector.

LC assay for nucleotides and nucleotide sugars was performed on a LC-20AR system (Shimadzu). The sample (20 μl) was separated on a Shim-pack GIST C18 column (Shimadzu, 5 μm, 4.6 mm × 250 mm). Mobile phase A was 20 mM phosphate buffer (pH = 5.8) in water, and mobile phase B was methanol. The flow rate was set as 1 ml/min. The elution was under an isocratic condition with 95% A for 10 min. Nucleotides and nucleotide sugars were detected with absorbance at 260 nm.

**LC-ESI-MS/MS to analyze metabolite profiling in rice seeds**

The wild-type and *xanP*-expressing transgenic rice seeds were ground into powder and incubated with methanol/acetonitrile/water (v/v/v = 2/2/1) solution at 4℃ with supersonic for 30 min. After [standing at -20 ℃ for](https://context.reverso.net/translation/english-chinese/standing+for) 10 min, the mixture was centrifuged at 14,000g at 4℃ for 20 min. The supernatant was dried by vacuum and the powder was dissolved in 100 μl of acetonitrile/water (v/v = 1/1) by vortexing. After centrifugation with 14,000 g at 4 ℃ for 15 min, the supernatant was subject to LC-MS. LC-MS was performed on an Agilent 1290 infinity LC system (Agilent Technologies), coupled to an AB Sciex 6600 triple TOF mass spectrometer (AB Sciex). During the assay, the samples were stored in the automatic sampler at 4℃. The extracts (2 μl) were injected into an ACQUITY UPLC BEH C-18 column (1.7 μm, 2.1 mm × 100 mm, Waters Corp., Milford, MA). Column oven temperature was keeping at 40℃. Mobile phase A was 25 mM ammonium acetate and 0.5% formic acid in water, and mobile phase B was methanol. Gradient elution was performed as follows: 0 - 0.5 min, 5% B; 0.5 -10 min, 5 - 100% B; 10.0 - 12.0 min, 100 % B; 12.0 - 12.1 min, 100 - 5 % B; 12.1 - 16 min, 5 % B. ESI was operated in positive and negative ion modes with parameters as follows: source temperature, 600℃; ionspary voltage floating, ± 5500 V; TOF MS scan range, *m/z* 60 - 1000. MS2 data were acquired by means of information dependent acquisition in a high sensitivity mode. The parameters were set as: exclude isotopes within 4 Da, and 10 candidate ions to monitor per cycle. Declustering potential was set at ± 60 V (positive and negative ion modes) and collision energy was 35 ± 15 eV. Raw data were transformed to mzXML files through ProteoWizard software (<http://proteowizard.sourceforge.net/>) and analyzed with MS-DAIL software (*34*).

**In vitro xanthosan synthesis with immunoprecipitated AvrBs2-FLAG**

The transgenic rice seedlings expressing AvrBs2-FLAG and its mutant proteins were ground into powder in liquid nitrogen. Total proteins were extracted after the powder was incubated in the extraction buffer [50 mM Tris-Cl, pH= 7.5, 150 mM NaCl, 1% NP-40 and 2 × protease inhibitor cocktail (MedChemExpress, HY-K0010) on ice bath for 30 min. After centrifugation at 12,000 g for 10 min at 4 ℃, 1.4 mL of the supernatant was incubated with 25 μL of anti-FLAG® M2 Affinity Gel (Sigma-Aldrich, A2220) for 2 h. The beads were spun down by centrifugation at 800 g for 1 min at 4 ℃, and were washed twice with the extraction buffer. Xanthosan was synthesized in a 100 μL mixture, including 25 μL of protein-bound beads, 1 mM UDP-α-D-Gal or UDP-α-D-Glc, 50 mM Tris-HCl, pH = 7.5, 150 mM NaCl and 10 mM MgCl_2_. After incubation on a rotary shaker for 4 h at 28 ℃, the mixtures were centrifuged at 800 g for 1 min at 4 ℃, and 70 μL of the supernatant was collected and mixed with 700 μl ethanol to precipitate the products at -20℃ overnight. The products were dissolved in ddH_2_O and were then detected with TLC and HILIC-ESI-MS as described above. AvrBs2-FLAG and its mutant proteins bound to the beads were detected by immunoblotting with a monoclonal HRP conjugated anti-FLAG antibody (Sigma-Aldrich, A8592) (1:5,000).

**Xanthosan uptake assay**

Overnight-cultured *Xoc* cells were collected by centrifugation at 4,000 rpm for 1 min. The cultures were washed and re-suspended with NB liquid media to a cell density of OD_600_ = 2.5. Cell suspensions (400 μl) were incubated with 1 mM of xanthosan and further cultured in a 24-well plate with shaking at 140 rpm at 28°C. The cultures (30 μl) were collected at the indicated time-points and were subject to centrifugation at 10,000 g for 1 min. Xanthosan in the supernatants was detected by TLC assays and quantified by band intensities with Photoshop.

To detect xanthosan inside bacterial cells, cell pellets were collected from 1ml of culture by centrifugation at 10,000 g after incubation with xanthosan for 0 and 12 h. After washing with ddH_2_O twice, cell pellets were used to extract the metabolites sequentially with 200 μl of 50% phenol and ethanol as described above. The purified metabolites were dissolved in ddH_2_O and analyzed by TLC.

**Bacterial growth assay**

Overnight-cultured *Xoc* cells were spun down by centrifugation at 4,000 rpm for 1 min. The cultures were washed three times and re-suspended with XVM2 minimal medium (20 mM NaCl, 10 mM (NH_4_)_2_SO_4_, 5 mM MgSO_4_, 1 mM CaCl_2_, 0.16 mM KH_2_PO_4_, 0.32 mM K_2_HPO_4_, 0.01 mM FeSO_4_, 0.03% casamino acids, pH 6.7) (*35*) to a cell density of OD_600_ = 0.5. Cell suspension was diluted (1:25 dilution) in XVM2 minimal medium supplemented with 0.05 mM sucrose, 5 mM xanthosan or both. Alternatively, cell suspensions were diluted (1:10 dilution) in XVM2 minimal medium supplied with 5 mM sucrose, 5 mM D-galactose or ddH_2_O as mock. Bacterial growth was measured in a Bioscreen C automatic growth analyzer at 28°C.

**Identification of *xanT*-*xanP* polycistron by RT-PCR**

Total RNAs were isolated from overnight-cultured *Xoc* cells using an EASYspin Plus Bacterial RNA Extraction kit (Aidlab Biotechnologies Co., Ltd, Beijing). After removing DNA contamination in extracted RNAs using DNase I, cDNAs were reversely transcribed with a TRUEscript RT Kit (Aidlab Biotechnologies). A fragment containing *xanT* and *xanP* was amplified by PCR with cDNAs as template. Genomic DNA and total RNAs were used as templates for PCR amplification as positive and negative controls, respectively.

**Measurement of ion leakage in *Xoc*-infected rice leaves**

Overnight-cultured *Xoc* cells were collected by centrifugation at 5,000 g for 10 min and were re-suspended with 10 mM MgCl_2_. The bacterial suspension (OD_600_ = 0.5) or 10 mM MgCl_2_ (mock) was infiltrated into rice leaves. At the indicated time points post infiltration, leaf segments (4-cm length) were detached and incubated in 3 mL of deionized water. The conductivity caused by ion leakage was measured at 6 h post incubation with a conductivity meter (DDSJ-318T, Leici, Shanghai, China) and recorded as C_l_. The samples were then boiled for 2 min to release cytosolic ions from leaf segments. After cooling to room temperature, the conductivity was measured again and recorded as C_t_. The relative conductivity was calculated with the formula: C_l_ / C_t_ × 100%.

**Transgenic constructs and plant transformation**

The IE-*avrBs2* transgenic rice line with DEX-inducible expression of AvrBs2 was generated previously (*8*). For DEX-inducible expression of AvrBs2^H319A^, pUC19-3*5Spro:avrBs2^H319A^*-*3×FLAG* was generated by site-directed mutagenesis using pUC19-*35Spro:avrBs2*-*3×FLAG* as a template (*8*). The *avrBs2^H319A^*-*3×FLAG* fragment was released from the plasmid by *Xho* I and *Spe* I and re-ligated into the DEX-inducible expression vector pTA7001. For ectopic expression of *xanP* and *xanP^M^* in rice, ORFs were amplified from pET28a-*xanP* and pET28a-*xanP^M1^* vectors, respectively, and were then sub-cloned into pC1305 (*36*). After confirmation by sequencing, these constructs were transformed into *A. tumefaciens* strain EHA105 through the freeze-thaw method (*37*). Transgenic rice plants were generated through *Agrobacterium*-mediated transformation.

**qRT-PCR analysis**

To detect the expression of *avrBs2*, *xanT* and *xanP* during *Xoc* infection, overnight-cultured *Xoc* strain RS105 was adjusted to a cell density of OD_600_ = 0.3 after washing with 10 mM MgCl_2_ twice and was pressure-inoculated into the leaves of 6-week-old rice plants. The leaves were collected at 0 and 2 dpi to extract total RNAs and cDNAs were reversely transcribed with a TRUEscript RT Kit. Quantitative RT-PCR (qRT-PCR) was performed using a QuantStudio^TM^ 6 Flex Real-Time PCR System (Applied Biosystems, Carlsbad, CA). The gene expression levels were calculated based on three biological replicates. The primers used for qRT-PCR are listed in Table S1.

**Phylogenetic tree construction**

AvrBs2 homologs were identified by PSI-BLAST (*38*). The phylogenetic tree of AvrBs2 homologs was constructed by maximum likelihood method using MEGA7 (*39*). The phylogenetic relationship among *Xanthomonas* strains was calculated by OrthoFinder version 2.5.2 (*40*), and the phylogenetic tree was generated by MEGA7.

**Protein 3D structure prediction**

The three-dimensional (3D) structures of AvrBs2 and XanP were predicted with AlphaFold and RoseTTAFold, respectively (*41*, *42*). The 3D homology structure of XanT was predicted with SWISS-Model (*43*). The graphs were generated with ChimeraX 1.3 software (*44*, *45*).

**Quantification and statistical analysis**

The data in each figure are from representative experiments that were independently repeated at least three times. Statistical analyses were performed with two-sided *t*-test, or one-way ANOVA, following multiple comparisons of means with Tukey’s honest significance test (α = 0.05).


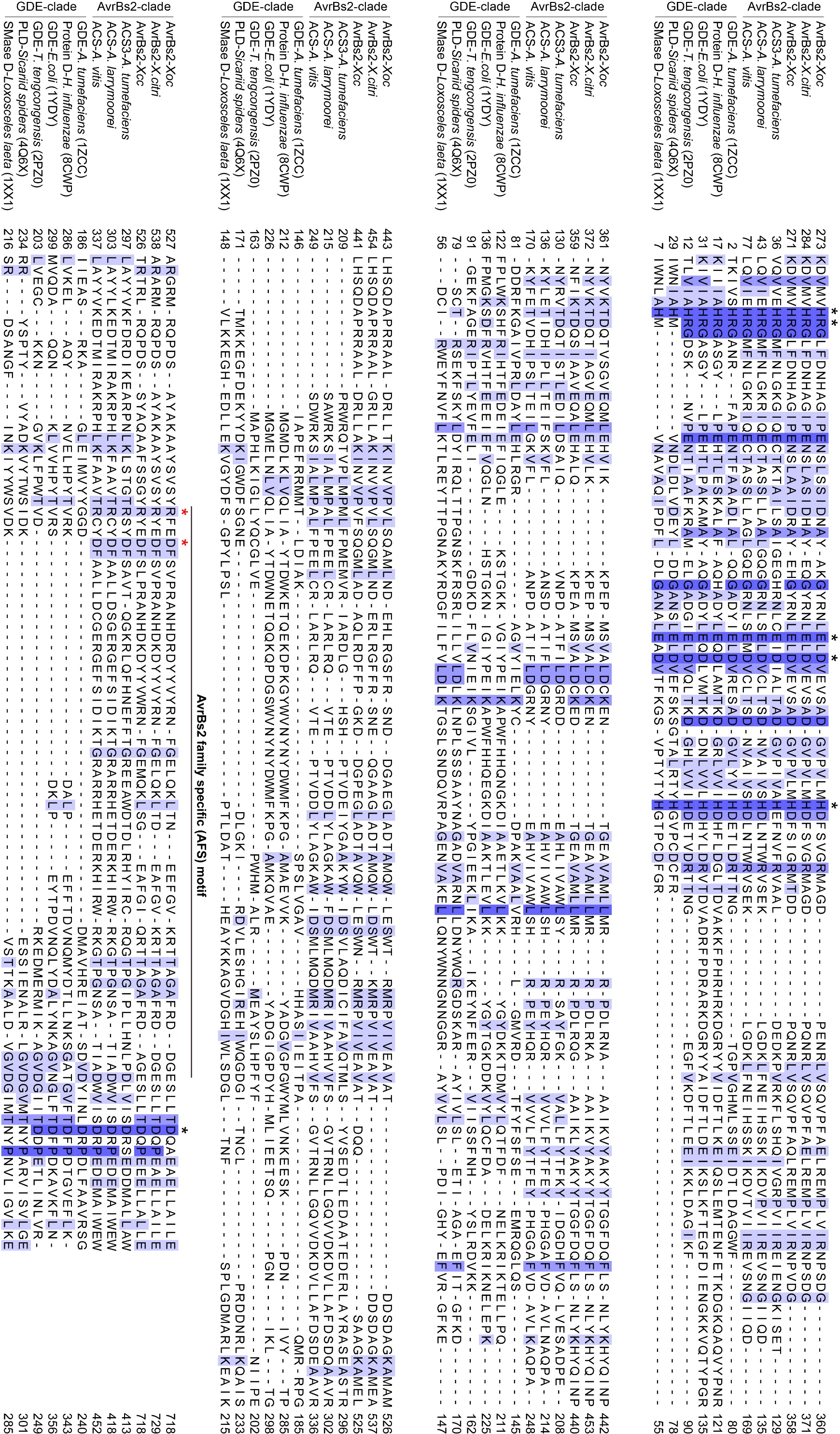


**Fig. S1. Amino-acid sequence alignment of putative catalytic domains in AvrBs2 clade proteins and canonical GDEs.** Sequence alignment was generated with PROMALS3D program based on comparison between the crystal structures of GDEs and predicted structures of AvrBs2-clade proteins by AlphaFold. The only GDE domains of AvrBs2 and its homologs in Fig. 1C were shown in the alignment. The AvrBs2-family specific (AFS) motif is indicated with a line. The conserved amino-acid residues are indicated by asterisks.


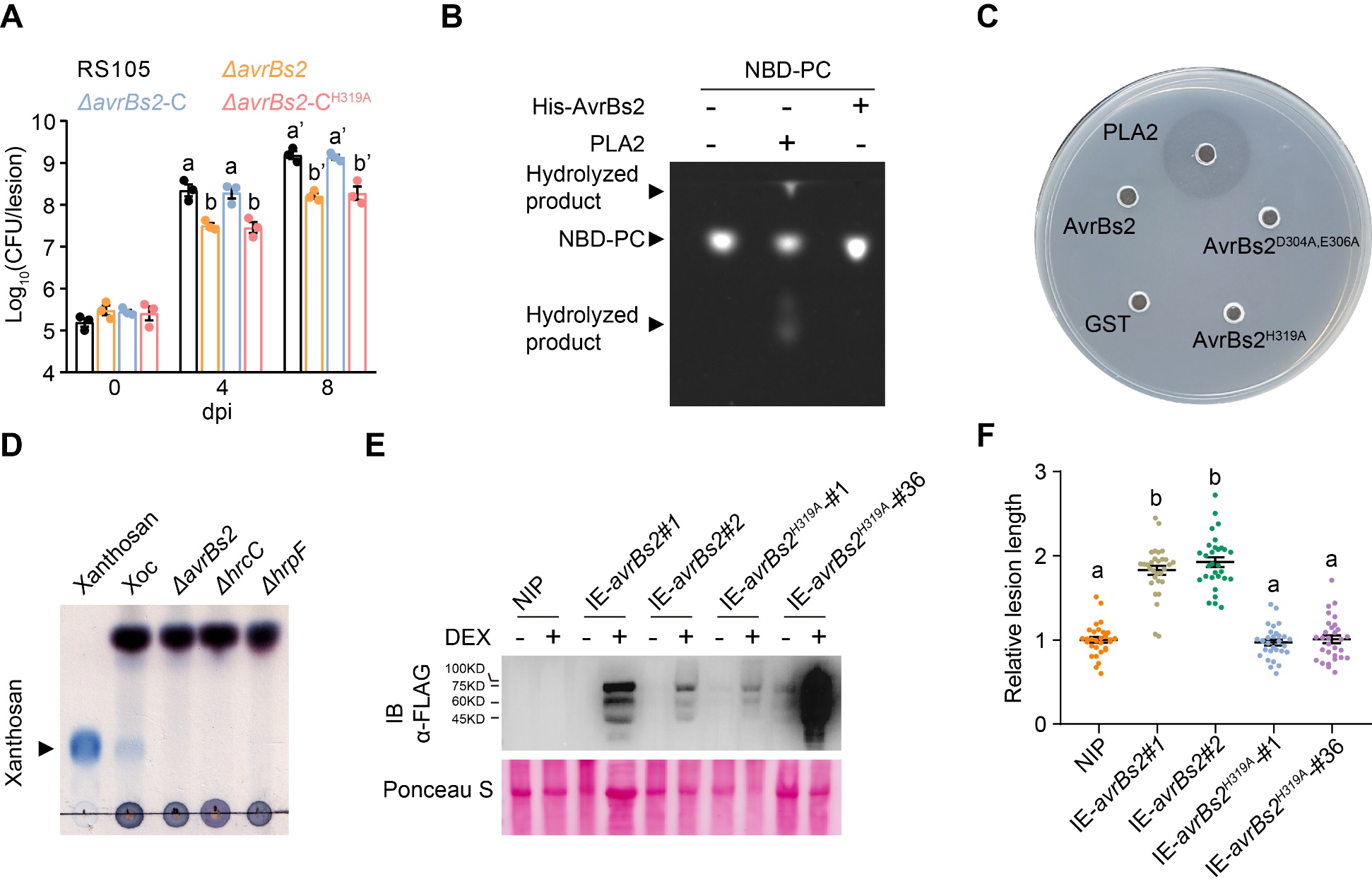


**Fig. S2. AvrBs2 is essential for *Xoc* virulence and xanthosan biosynthesis, but does not hydrolyze phospholipids.** (**A**) Bacterial population sizes in the wild-type rice leaves at 0-, 4-, and 8-day post inoculation with different *Xoc* strains. dpi, days post inoculation. Data are shown as mean ± standard error (SE, n = 3). Statistically significant difference in bacterial population size was revealed by one-way ANOVA, Tukey’s honest significance test with α = 0.05. (**B**) No phospholipid hydrolysis activity was detected under the tested conditions by TLC analysis for recombinant AvrBs2 purified from *E. coli.* Fluorescent NBD-PC was used as substrates. PLA2, a commercial snake phospholipase A2, was used as a positive control. (**C**) A phospholipase assay on egg yolk plates. GST-AvrBs2 and its variants were expressed and purified from *E. coli.* PLA2 and GST were used as positive and negative controls, respectively. (**D**) TLC analysis to detect xanthosan in rice leaves after infection with the wild-type *Xoc*, *ΔavrBs2* and the T3SS-deficient mutant strains *ΔhrcC* and *ΔhrpF*. Representative images from 3 independent experiments are shown. (**E**) The DEX-induced expression levels of AvrBs2-FLAG and AvrBs2^H319A^-FLAG as detected by immunoblotting in IE-*avrBs2* and IE-*avrBs2^H319A^* transgenic rice plants, respectively. These transgenic lines were treated with DEX (30 μM in 0.01% Silwet L77) or 0.01% Silwet L77 (Mock) before protein extraction. Upper panels, AvrBs2-FLAG and AvrBs2^H319A^-FLAG were detected by immunoblotting (IB) with an anti-FLAG antibody in the indicated transgenic lines. Lower panels, protein loading is indicated by Ponceau S staining. WT, wild-type plant; α-FLAG, anti-FLAG antibody. (**F**) The relative disease lesion lengths on the wild-type, IE-*avrBs2* and IE-*avrBs2^H319A^* transgenic rice leaves caused by inoculation with the *ΔavrBs2* mutant strain. The lengths of disease lesions on transgenic rice leaves are normalized to those in the wild-type plant leaves. Data from three independent assays are shown. Different letters (a-b) indicate statistically significant differences (One-way ANOVA, Tukey’s honest significance test with α = 0.05).


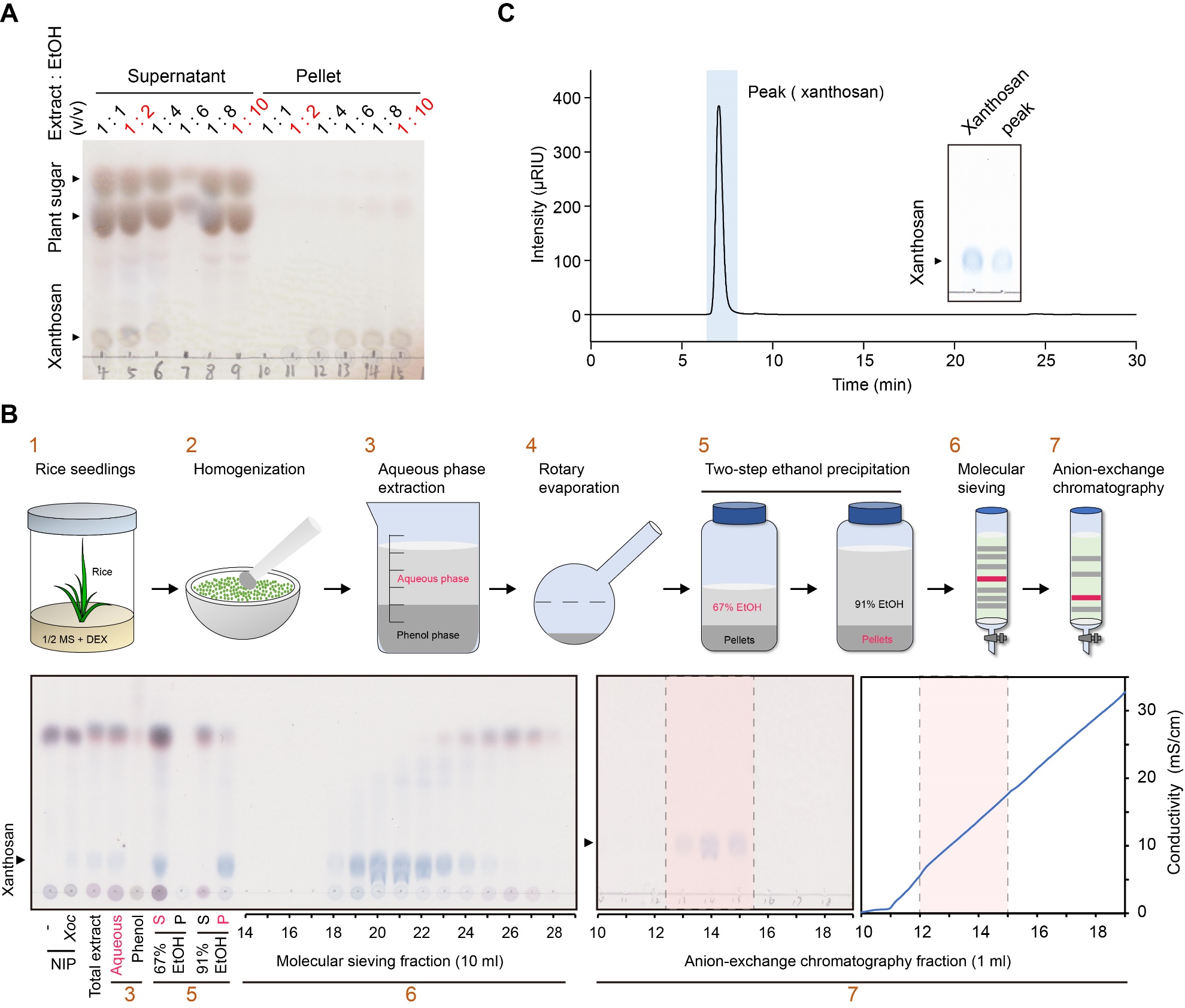


**Fig. S3. Purification of xanthosan from *avrBs2* transgenic plants.** (**A**) Purification of xanthosan using a gradient concentration of ethanol. Crude xanthosan extract was concentrated and was then mixed with gradient concentrations of ethanol. The pellet and supernatant were analyzed by TLC. (**B**) The procedure to purify xanthosan from *avrBs2*-expressing rice plants. The upper schematic diagram showed the steps for xanthosan purification. The purified products were detected by TLC assays (lower graph). S, supernatant; P, pellet. **(C)** The purity of purified xanthosan as detected by liquid chromatography. The peak was monitored with refractive index detector. Fraction in the peak was collected and detected with TLC as shown in the graph.


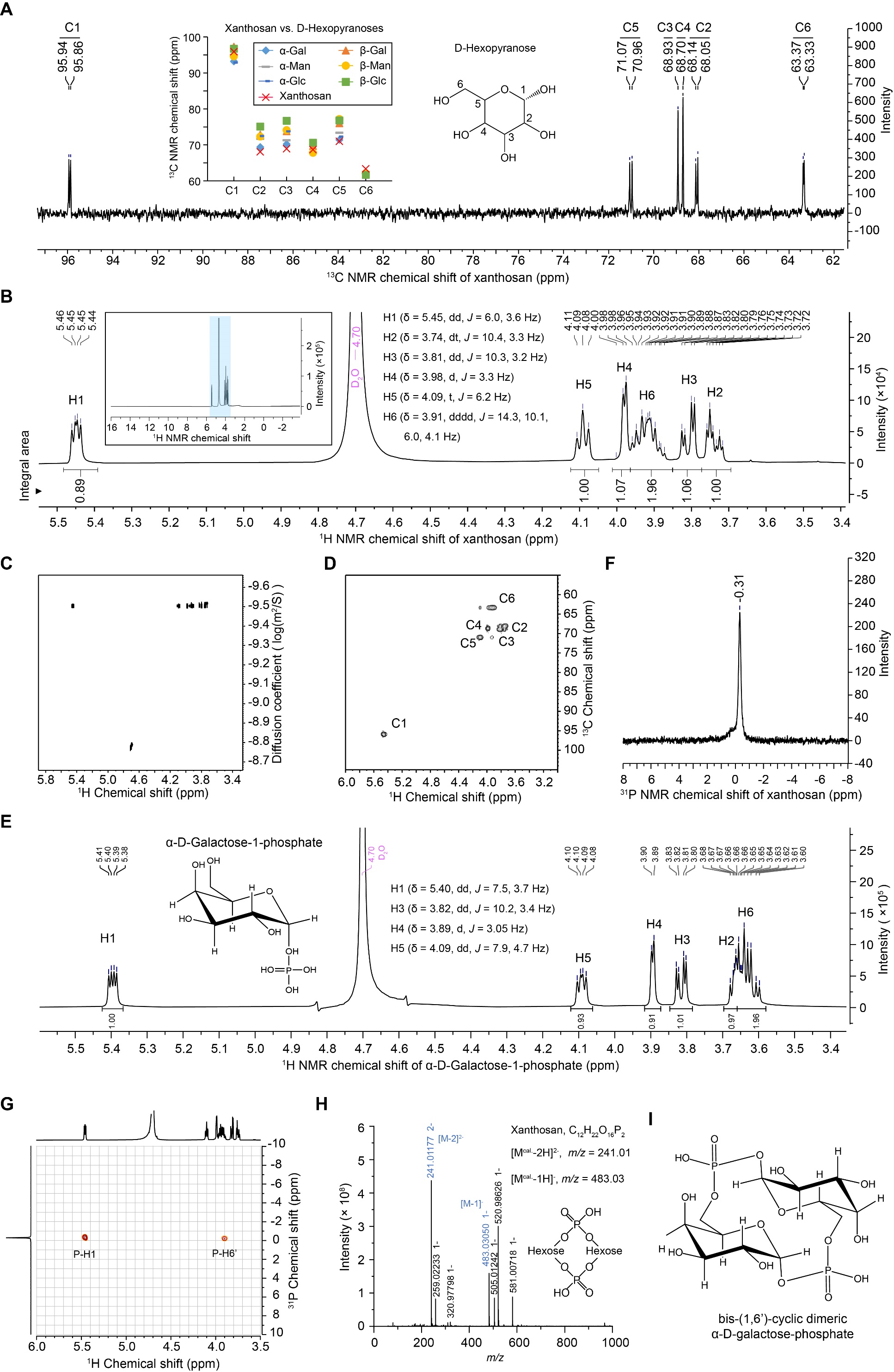


**Fig. S4. Determination of the xanthosan structure.** (**A**) 1D ^13^C-NMR spectrum of xanthosan in D_2_O and comparison of ^13^C-NMR chemical shifts of xanthosan and D-hexopyranoses. The assignment of the ^13^C resonances of xanthosan was determined based on the ^1^H-^13^C-HSQC-NMR in Fig. S4D. In the chart, ^13^C chemical shifts of D-hexopyranoses are from a published literature (*15*). (**B**) 1D ^1^H-NMR spectroscopy of xanthosan in D_2_O. The full ^1^H NMR spectrum was shown in the box, and the proton signal-containing region was expanded and shown as the main spectrum. The *J*-coupling constants (H1 - H6) were listed. (**C**) ^1^H-DOSY-NMR spectroscopy of xanthosan in D_2_O. (**D**) ^1^H-^13^C-HSQC-NMR spectroscopy of xanthosan in D_2_O. (**E**) The ^1^H-NMR spectrum of α-D-galactose-1-phosphate. The *J*-coupling constants (H1, H3, H4 and H5) were listed. (**F**) The ^31^P-NMR spectrum of xanthosan in D_2_O. (**G**) ^1^H-^31^P-HSQC-NMR spectrum of xanthosan in D_2_O. (**H**) The molecular mass of xanthosan was determined by ESI-FT-ICR-MS under a negative ion mode. (**I**) The inferred structural formula of xanthosan.


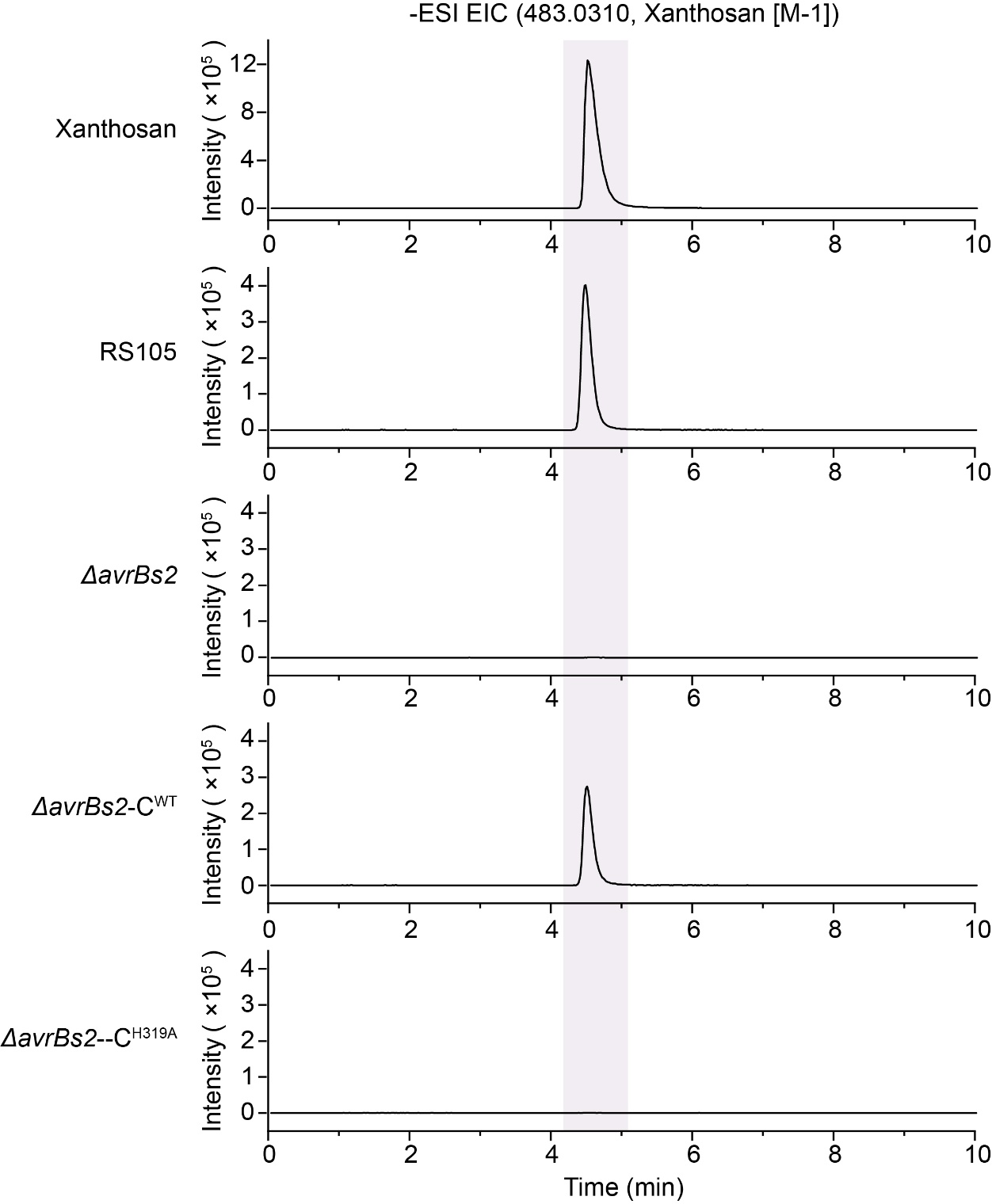


**Fig. S5. HILIC-ESI-MS assay to detect xanthosan generated in rice leaves infected by different *Xoc* strains.** HILIC-ESI-MS chromatogram traces corresponding to the ion extraction of bis-1,6-cyclic dimeric α-D-galactose-phosphate (theoretical mass [M-H]^-^ = 483.0310 Da; ion extraction: 483.0310 *m/z*) in purified xanthosan and total metabolite extracts from rice leaves infected by the indicated *Xoc* strains. Liquid chromatograph was conducted with an isocratic elution procedure described in methods.


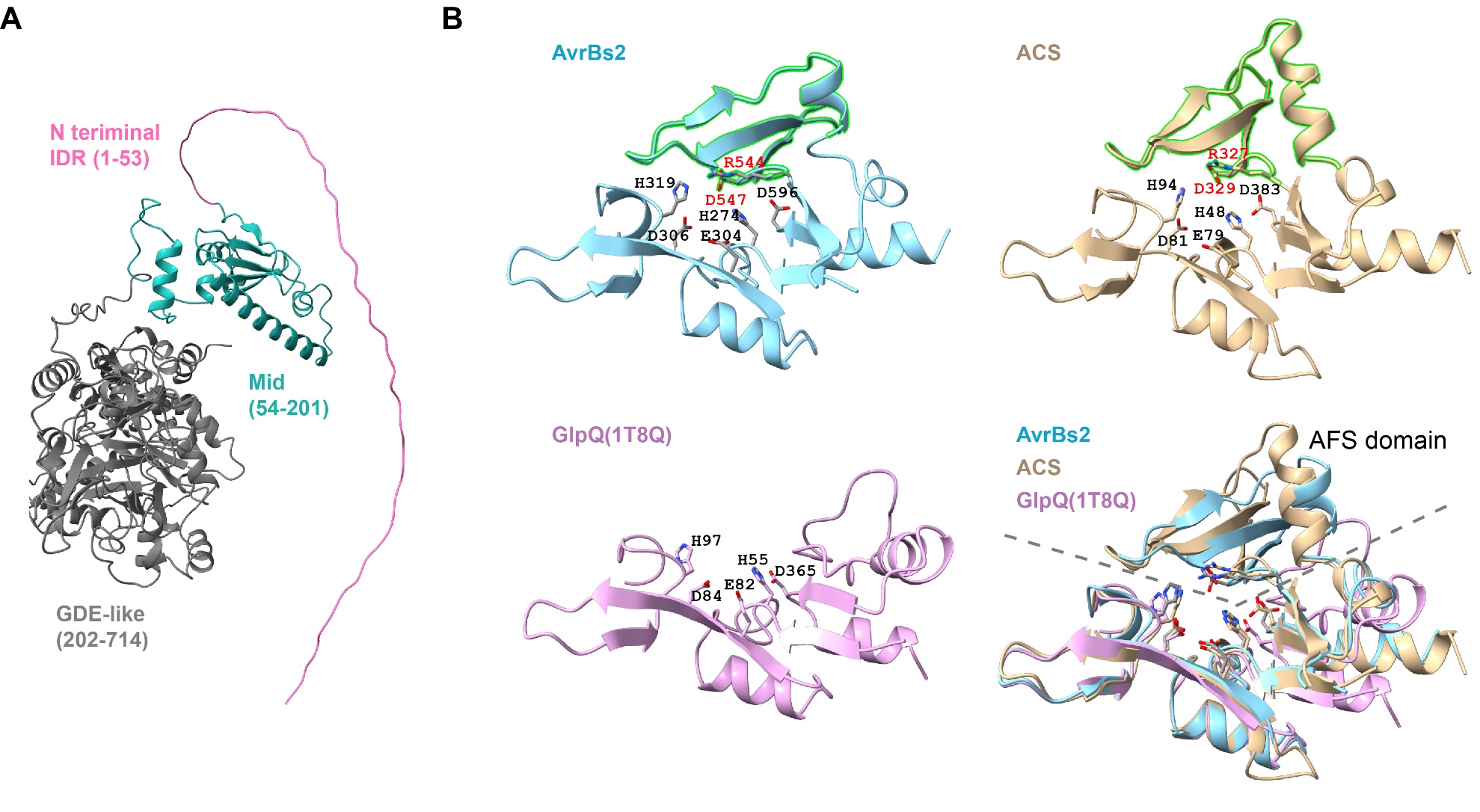


**Fig. S6. AvrBs2 contains a specific GDE-like catalytic domain.** (**A**) The predicted 3D structure of AvrBs2 by AlphaFold. (**B**) Comparison of catalytic pockets among AvrBs2, ACS and GlpQ. The AvrBs2-family specific motif is highlighted by green in AvrBs2 and ACS.


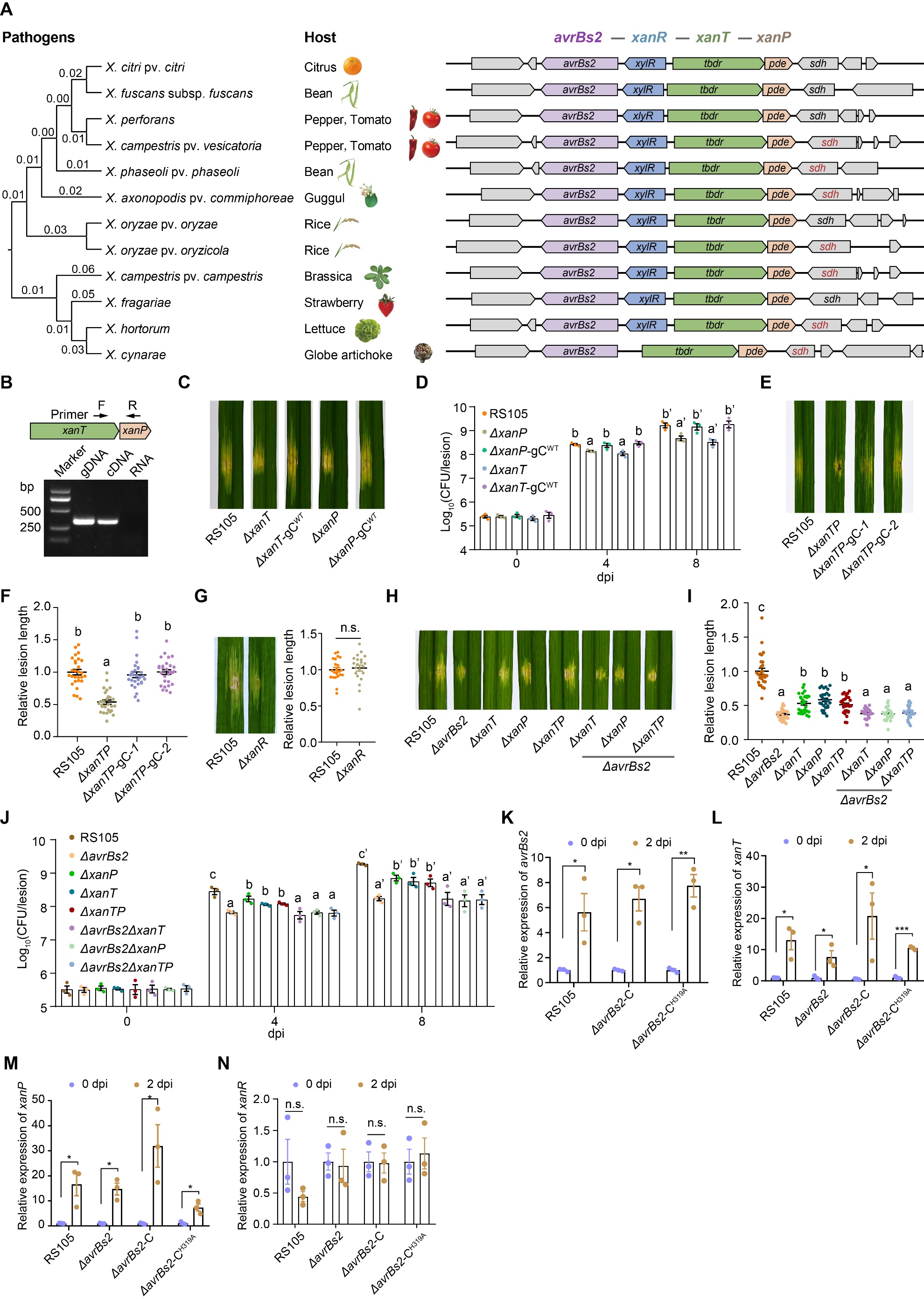


**Fig. S7. *avrBs2*, *xanT* and *xanP* form a virulence-related gene cluster in *Xoc*.** (**A**) A conserved gene cluster harboring *avrBs2*, *xanT* and *xanP* genes in *Xanthomonas* genus. (**B**) Confirmation of the *xanT*-*xanP* polycitron by RT-PCR. Total RNA and genomic DNA were extracted from overnight-cultured *Xoc* RS105 bacteria in NB medium. PCR fragment straddling *xanT* and *xanP* was amplified using *Xoc* cDNAs as template. PCR amplification using *Xoc* genomic DNA and total RNAs as templates was performed as positive and negative controls, respectively. (**C**) The images showing the disease lesions in Fig. 3A. (**D**) *Xoc* population sizes in rice leaves infected by *xanP* or *xanT* knockout mutant or their complemented *Xoc* strains at 0, 4 and 8 dpi. Data are shown as mean ± SE (n = 3). (**E**-**F**) Disease lesion lengths on rice leaves infected by the *xanTP* polycistron-deletion and complemented strains. WT, the wild-type strain; *ΔxanTP*, *xanTP* polycistron-deletion strain; *ΔxanTP*-gC-1 and -2, *ΔxanTP* complemented strains with the *xanT*-*xanP* genomic fragment. Data from three independent experiments are shown as mean ± SE. (**G**) Disease lesion lengths on rice leaves infected by the wild-type *Xoc* RS105 and an *xanR-*knockout strain. *ΔxanR*, a knockout mutant of *xanR* in *Xoc*. Data are shown as mean ± SE. (**H-I**) Disease lesion lengths on rice leaves infected by *avrBs2*, *xanP* or *xanT* knockout mutant strain. Data from three independent experiments are shown as mean ± SE. (**J**) *Xoc* population sizes in rice leaves infected by *Xoc* strains at 0, 4 and 8 dpi. Data are shown as mean ± SE (n=3). (**K-N**) The transcript levels of *avrBs2*, *xanT*, *xanP* and *xanR* in infected rice leaves at 2 days post *Xoc* inoculation as detected by qRT-PCR. The gene expression level was normalized by comparing with that at 0 dpi. 16S rDNA was used as an internal reference. Data are shown as mean ± SE (n=3). In **D**, **F**, **I** and **J**, different letters indicate statistically significant differences among different strains (one-way ANOVA, Tukey’s honest significance test with α = 0.05). In **G** and **K-N**, a statistically significant difference was revealed by Student’s *t*-test (*, *P* < 0.05；**, *P* < 0.01；***, *P* < 0.001); n.s., no significant difference.


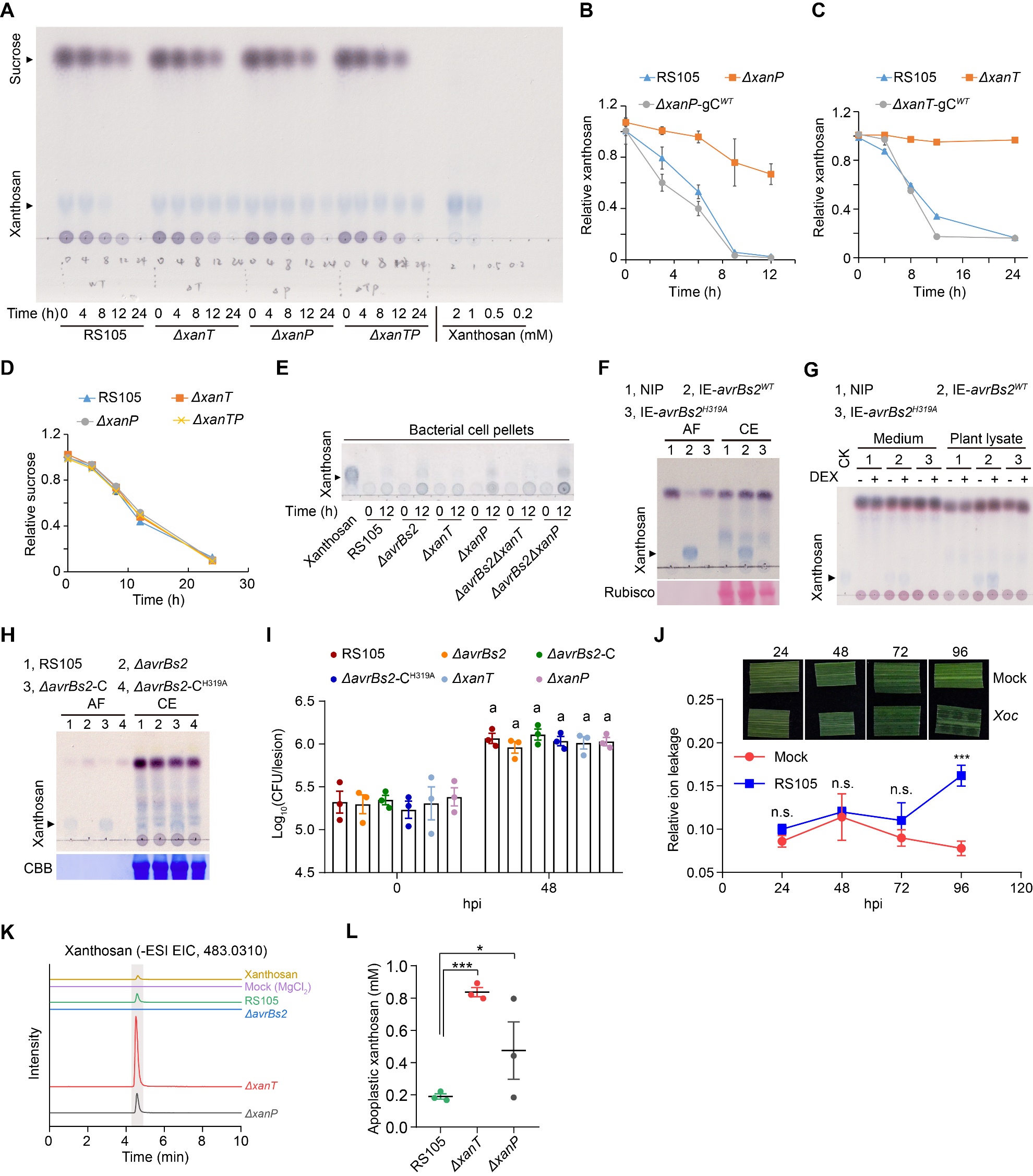


**Fig S8. XanT and XanP are essential for uptake of xanthosan into bacterial cells from plant apoplastic fluids.** (**A**) The original image of TLC assay in Fig. 3C. Different concentrations of purified xanthosan were loaded onto a TLC plate as reference to quantify xanthosan in the samples. (**B**-**C**) Xanthosan uptake assays in the *xanP-* and *xanT*-knockout and complemented strains as described in Fig. 3C. Data shown as mean ± SE (n = 3). The strains used in these experiments were described in Fig. 3A. (**D**) Sucrose uptake assay as described in Fig. 3C. The amount of sucrose remaining in the culture medium was detected by TLC as a control and was then quantified from three biological replicates. Data are shown as mean ± SE. (**E**) The amount of xanthosan accumulated in the cells of different *Xoc* strains. Different *Xoc* strains were incubated with xanthosan-containing NB medium as described in Fig. 3C. Xanthosan inside *Xoc* cells was extracted at the indicated time points and analyzed by TLC. (**F**) The amount of xanthosan in apoplastic fluids of *avrBs2*-expressing rice leaves. Apoplastic fluids were isolated from the leaves of the wild-type, *IE*-*avrBs2* and *IE*-*avrBs2^H319A^* transgenic rice plants through the infiltration-centrifugation method. Xanthosan levels in apoplastic fluids and cytosolic extracts were detected by TLC. Cytosolic contamination in apoplastic fluids was detected by Ponceau S staining of Rubisco proteins. AF, apoplastic fluids; CE, cytosolic extracts. (**G**) The release of xanthosan into the culture media from AvrBs2-expressing rice seedlings. The wild-type and *avrBs2-*/*avrBs2^H319A^*-expressing seedlings (7-day-old) were cultured in 1/2 × MS medium containing 30 μM DEX or mock for 48 h. Xanthosan levels in the culture medium and plant lysates were detected by TLC. CK, purified xanthosan. (**H**) Detection of xanthosan in apoplastic fluids of rice leaves infected by different *Xoc* strains. Apoplastic fluids were isolated from the rice leaves at 48 h post inoculation of *Xoc* strains. Xanthosan levels in apoplastic fluids and cytosolic extracts were detected with TLC. Cytosolic protein leakage in apoplastic fluids was minimal, as detected by staining Rubisco proteins with Coomassie Brilliant Blue. AF, Apoplastic fluids; CE, cytosolic extracts. (**I**) Population sizes of different *Xoc* strains in rice leaves at 0 and 48 h post infiltration. No significant difference was detected among the tested strains (one-way ANOVA, Tukey’s honest significance test with α = 0.05). (**J**) Time course assay of ion leakage in mock (MgCl_2_)- or *Xoc*-infiltrated rice leaves. Upper images showed water soaking in infected leaves at indicated time-points post infiltration. Lower chart showed the relative ion leakage of infected leaves. Data are shown as mean ± SE (n = 5). (**K**-**L**) HILIC-ESI-MS assays to detect xanthosan in the apoplastic fluids of rice leaves infected by different *Xoc* strains. Apoplastic fluids were isolated from rice leaves at 48 h after infiltration with mock solution (10 mM MgCl_2_) and *Xoc* strains. HILIC-ESI-MS chromatogram trace corresponding to the ion extraction of purified xanthosan ([M-H]^-^, ion extraction: 483.0310 *m/z*) or xanthosan in the apoplastic fluids of rice leaves (**K**). Quantification of xanthosan in the apoplastic fluids (**L**). Data are shown as mean ± SE (n = 3). In **J** and **L**, statistically significant differences were revealed by Student’s *t*-test (*, *P* < 0.05; ***, *P* < 0.001; n.s., no significant difference).


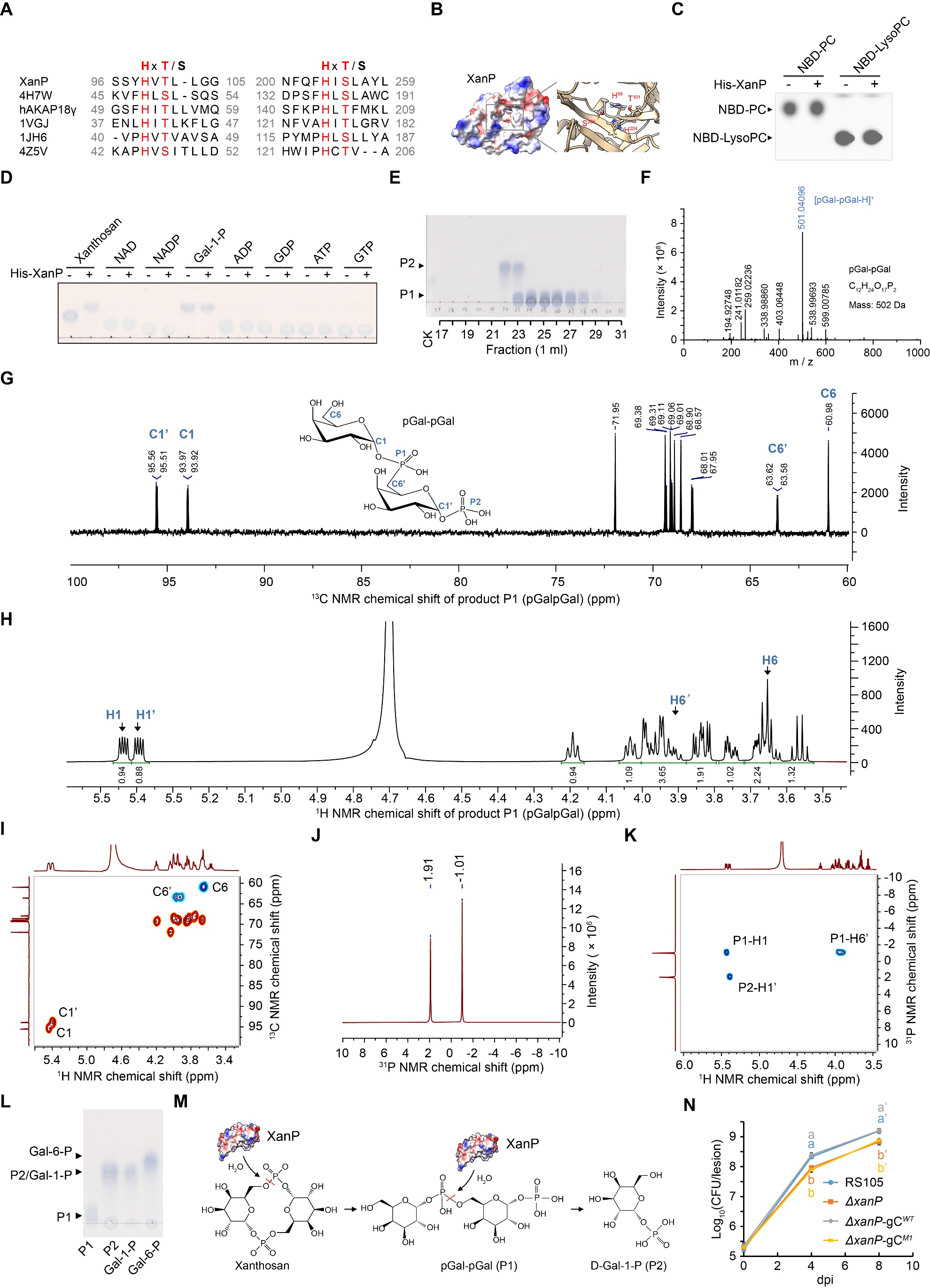


**Fig. S9. XanP is a xanthosan phosphodiesterase.** (**A**) Sequence alignment of the putative catalytic motifs in XanP and other 2H-phosphoesterases. (**B**) Structure modelling using RoseTTAFold to reveal the conserved “H×T/S” motifs in the catalytic pocket of XanP. (**C**-**D**) The substrate specificity assay of XanP. The compounds containing various phosphoester bonds were incubated with in vitro-purified XanP, and were then analyzed by TLC. (**E**) Anion-exchange chromatography to isolate the hydrolysates of xanthosan after incubation with XanP. The partially (P1) and completely (P2) hydrolytic products are indicated by arrowheads. CK, purified xanthosan. (**F**) The molecular mass of the partially hydrolytic product P1 of xanthosan as determined by ESI-FT-ICR-MS under a negative ion mode. **(G-I)** Determination of C-H linkages in the xanthosan hydrolysate based on ^13^C-NMR (**G**) ^1^H-NMR (**H**), and ^1^H-^13^C HSQC-NMR (**I**) spectra. (**J-K**) ^31^P-NMR (**J**) and ^1^H-^31^P HSQC-NMR (**K**) spectra of the xanthosan hydrolysate pGal-pGal. (**L**) TLC assay to identify the hydrolytic products of xanthosan. (**M**) A schematic diagram to show two steps of xanthosan cleavage by XanP. (**N**) Bacterial population sizes in infected rice leaves at the indicated time points after infiltration with *ΔxanP* and its complemented strains. Data are shown as mean ± SE (n = 3). *ΔxanP*-gC*^WT^* and *ΔxanP*-gC*^M1^*, *ΔxanP* strains genomically complemented with the full-length *xanP* and *xanP^H99A/T101A^* genes, respectively. Different letters indicate statistically significant differences among different strains (one-way ANOVA, Tukey’s honest significance test with α = 0.05).


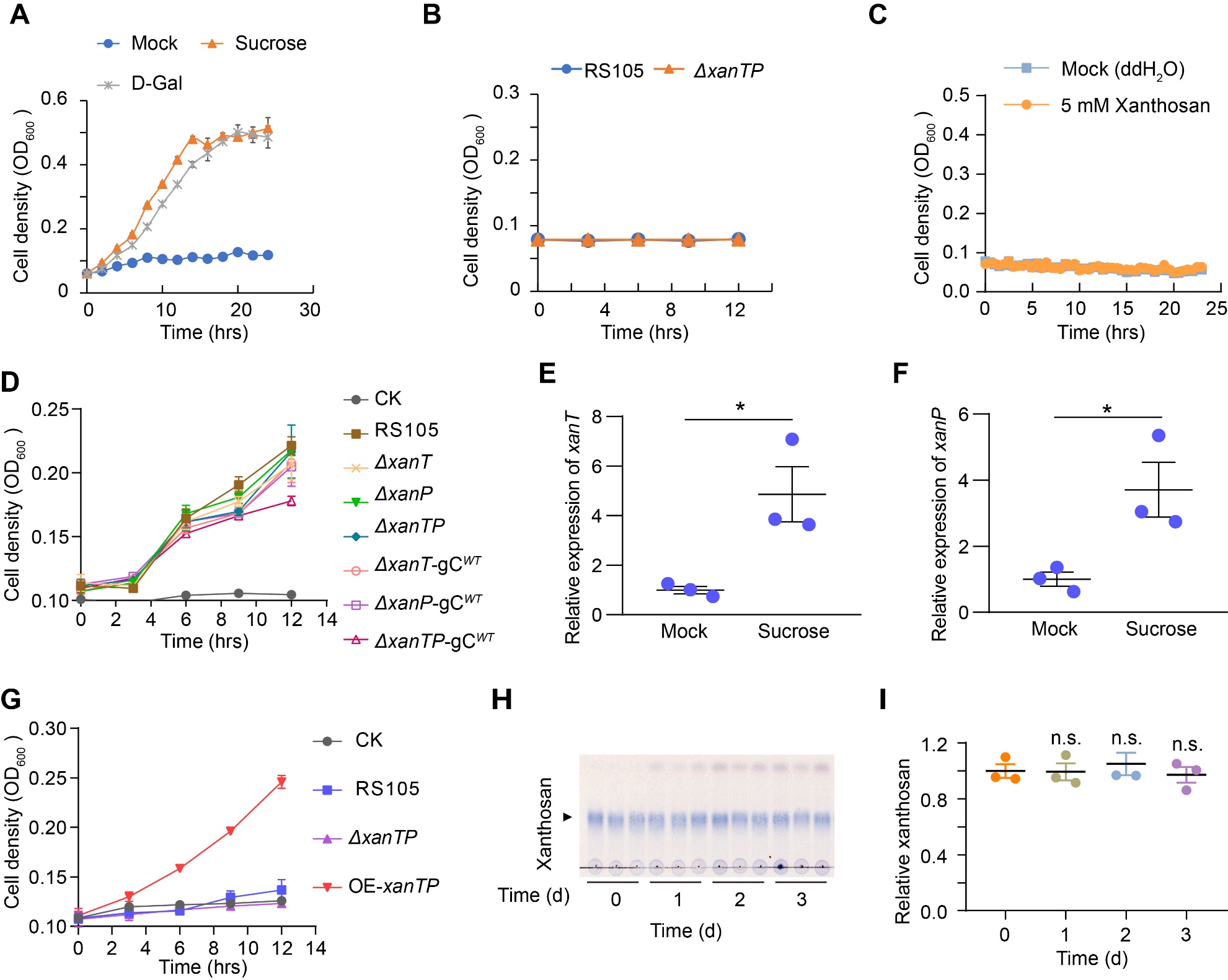


**Fig. S10. Xanthosan is a specific nutrient for *Xoc*.** (**A**) Growth curve assay of the *Xoc* strain RS105 in the modified XVM2 minimal medium containing 5 mM sucrose or 5 mM D-galactose (D-Gal) as sole carbon source or ddH_2_O as mock. (**B**) Growth curve assay of *Xoc* strains in the modified XVM2 minimal medium supplemented with 0.05 mM sucrose as sole carbon source. (**C**) Growth curve assay of the *Xoc* strain RS105 in the modified XVM2 minimal medium supplemented with 5 mM xanthosan as sole carbon source. (**D**) Growth curve assay of *Xoc* strains in the modified XVM2 minimal medium supplemented with 5 mM sucrose as sole carbon source. (**E-F**) RT-qPCR assay to detect the transcript levels of *xanT* (**E**) and *xanP* (**F**) in *Xoc* at 2 hours after incubation with mock (ddH_2_O) or 0.05 mM sucrose. The gene expression level was normalized by comparing with mock treatment. 16S rDNA was used as an internal reference. Data are shown as mean ± SE (n=3). Statistically significant difference was revealed by Student’s *t*-test (*, *P* < 0.05). (**G**) Growth curve assay of the *Xoc* strains in the XVM2 minimal medium supplemented with 5 mM xanthosan as sole carbon source. CK, ddH_2_O. CE-*xanTP*, a *ΔxanTP* complemented strain constitutively expressing *xanTP* gene. (**H-I**) The stability of xanthosan in *N. benthamiana* leaves. Leaf discs were collected to detect xanthosan content by TLC assay at different days post infiltration with xanthosan. n.s. indicates no significant difference in the content of xanthosan in *N. benthamiana* leaves at 0-3 days post infiltration (Student’s *t*-test, *P* < 0.05).


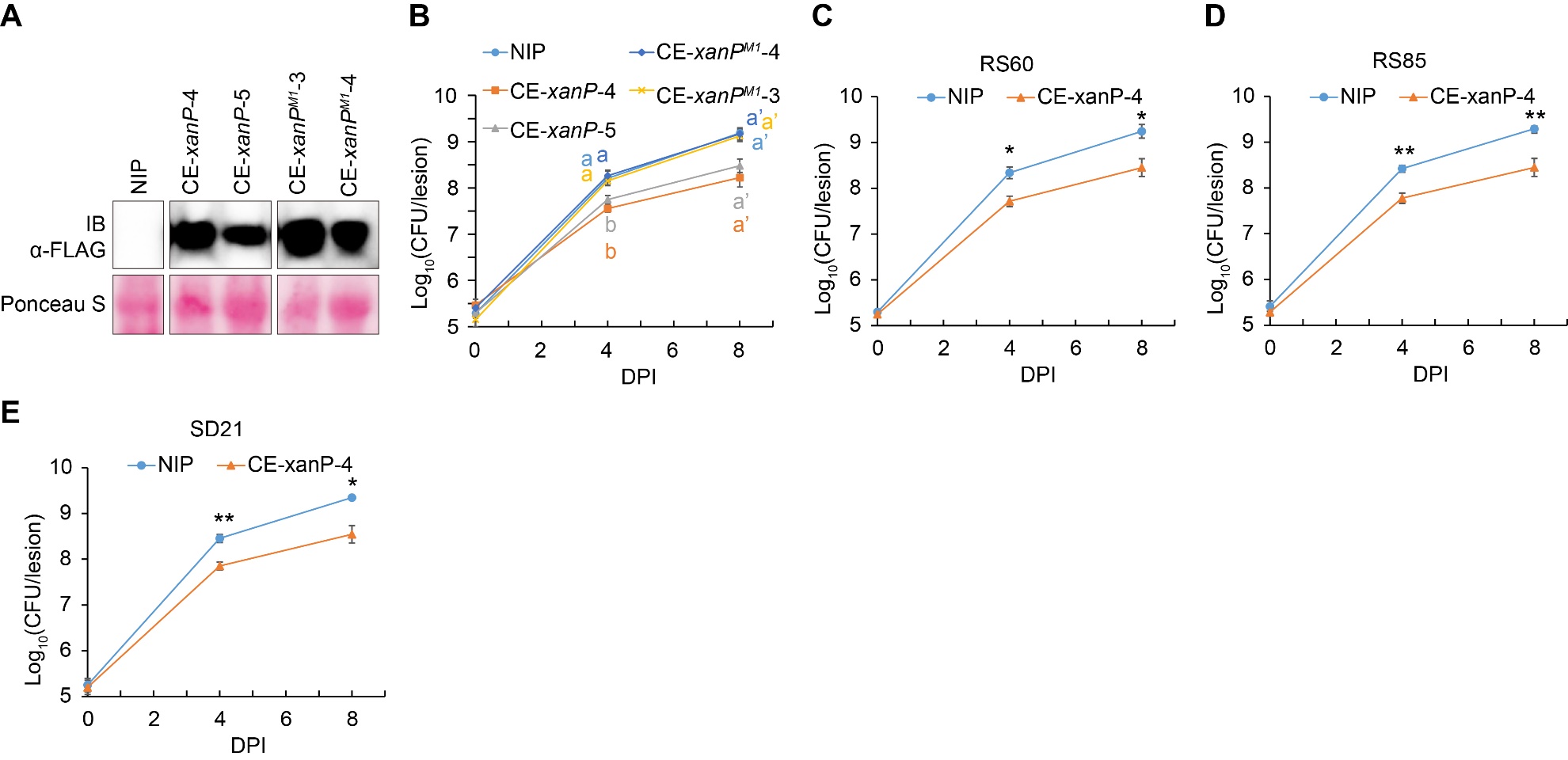


**Fig. S11. Ectopic expression of XanP confers enhanced resistance to bacterial leaf streak in rice.** (**A**) The expression levels of XanP-FLAG and XanP^H99A/T101A^-FLAG in the CE-*xanP* and CE-*xanP^M1^* transgenic rice lines transformed with pC1305-*xanP-FLAG* and pC1305-*xanP^M1^-FLAG*. CE, constitutive expression. Upper panel, XanP-FLAG and XanP^H99A/T101A^-FLAG were detected by immunoblotting with an anti-FLAG antibody in the transgenic lines. Lower panel, protein loading is indicated by Ponceau S staining. WT, the wild-type plant; α-FLAG, anti-FLAG antibody. (**B**-**E**) The population sizes of different *Xoc* strains in the wild-type, XanP- or XanP^M1^-expressing rice leaves after infection with the *Xoc* strains RS105 (**B**), RS60 (**C**), RS80 (**D**), and SD21 (**E**).


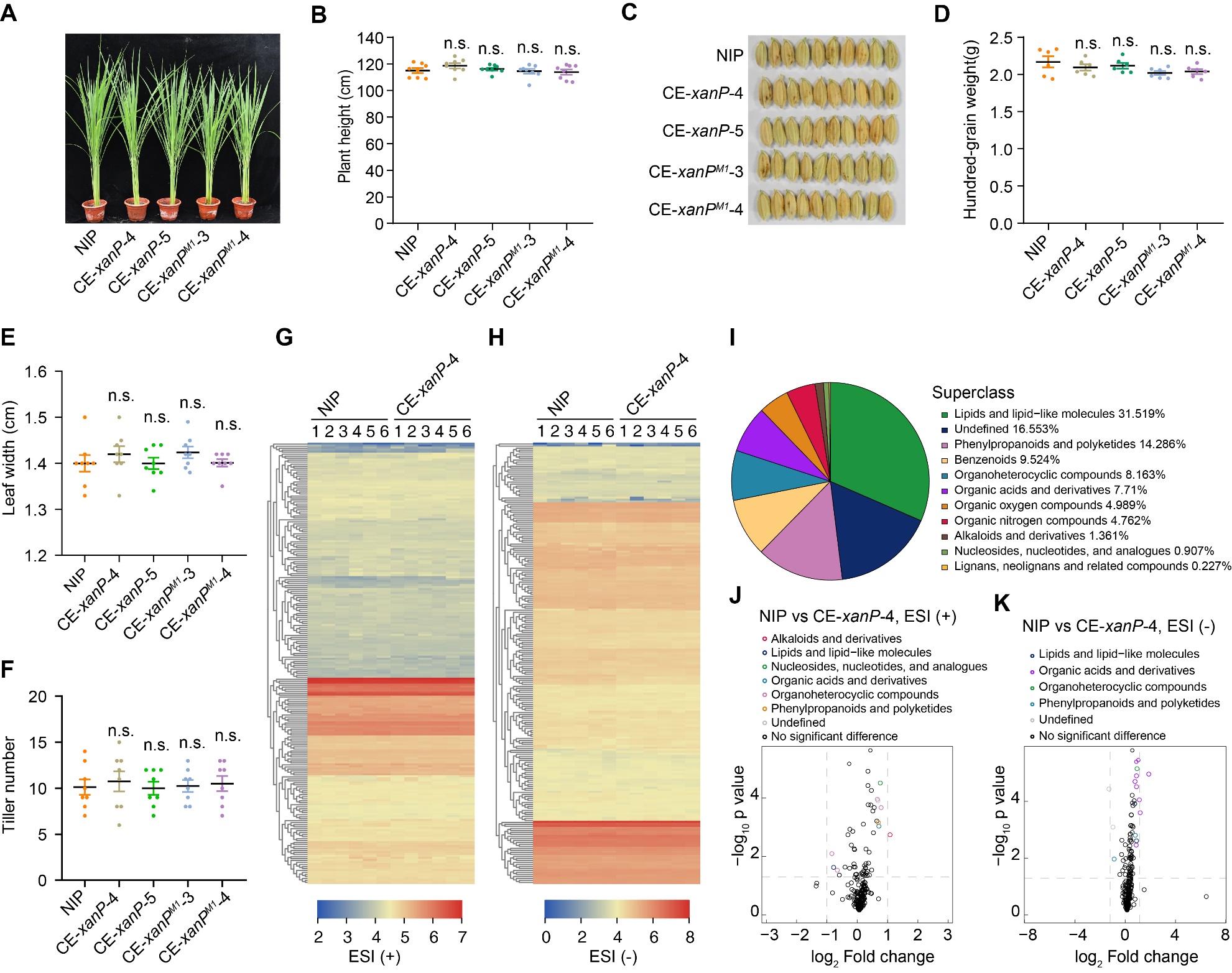


**Fig. S12. Ectopic expression of XanP has no influence on agronomic traits or metabolism in transgenic rice plants.** (**A**-**B**) The height of the wild-type, *xanP*- and *xanP^M1^*-expressing transgenic plants. The image showed 8-week-old rice plants (**A**). The chart exhibited the height of the wild-type and transgenic rice plants (**B**). (**C**-**F**) The size of matured rice seeds (**C**), the hundred-grain weight (**D**), leaf width (**E**), and tiller number (**F**) of the wild-type, *xanP*- and *xanP^M1^*-expressing transgenic plants. (**G**-**H**) Metabolome analysis of the wild-type and CE-*xanP*-4 transgenic rice seeds. Color bars from blue to red indicate the relative contents of metabolites. A total of 191 and 178 metabolites were annotated in all metabolites identified by LC-ESI-MS/MS in positive (**G**) and negative (**H**) ion models, respectively. (**I**) Classification of identified metabolites. (**J**-**K**) Volcano plots to indicate a consistent metabolite distribution between the wild-type and CE-*xanP*-4 transgenic rice seeds. Significant difference is judged by |log2 (Fold Change) | > 1 and *P* value < 0.05. In **B**, **D**, **E**, and **F**, data are shown as mean ± SE. n.s. indicates no significant difference in plant height, hundred-grain weight, leaf width, and tiller number among the wild-type, *xanP*- and *xanP^M1^*-expressing transgenic plants (one-way ANOVA, Tukey’s honest significance test with α = 0.05).


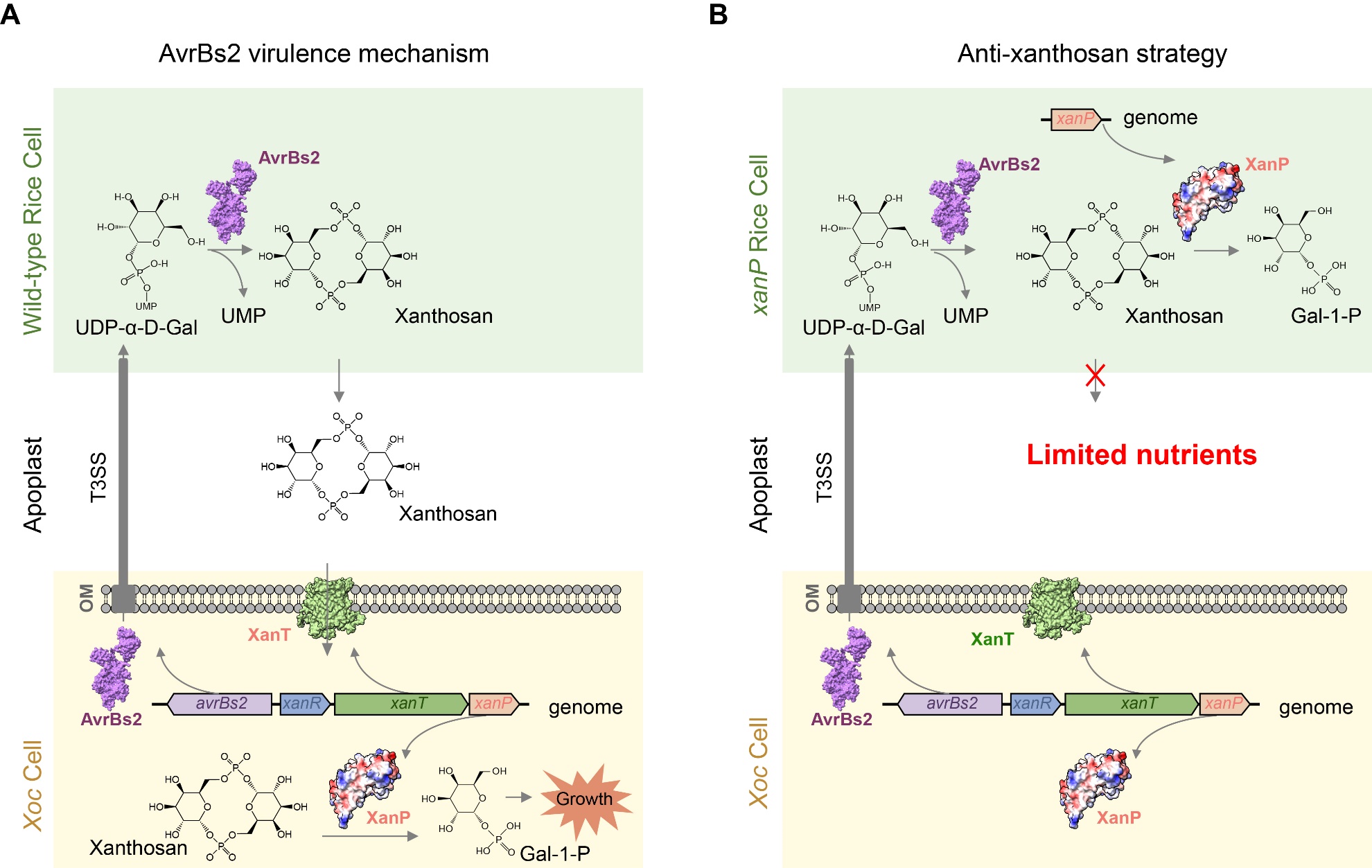


**Fig. S13. A proposed model for the xanthosan generation-uptake-utilization system in *Xoc* (A) and anti-xanthosan strategy to control plant *Xanthomonas* diseases (B).** (**A**) During *Xoc* infection, AvrBs2 is secreted into host cells to synthesize xanthosan using UDP-α-D-galactose. After xanthosan is released into host apoplastic spaces, the outer membrane transporter XanT in *Xoc* specifically transports xanthosan into bacterial cells, and the phosphodiesterase XanP hydrolyzes xanthosan for bacterial multiplication. (**B**) Ectopically expressed XanP in transgenic rice plants hydrolyzes AvrBs2-synthesized xanthosan to limit bacterial nutrient supply, thus resulting in enhanced resistance to *Xoc*.

**Table S1. Primer sequences in the research.**

| **primers** | **sequence(5'-3')** |
| --- | --- |
| avrBs2-H274A-F | TAGGAAAGGACGTGATGGTGGCTCGCGGGCTGTTCGATAACCA |
| avrBs2-H274A-R | TGGTTATCGAACAGCCCGCGAGCCACCATCACGTCCTTTCCTA |
| avrBs2-E304A/D306A-R | CCATCGGCACTGACTTCGACGGCCAGCGCCAGATT |
| avrBs2-E304A/D306A-F | ATCTGGCGCTGGCCGTCGAAGTCAGTGCCGATGG |
| avrBs2-H319A-F | CCGGTGTTGATGGCCGATTTCAGCGTGGG |
| avrBs2-H319A-R | CCCACGCTGAAATCGGCCATCAACACCGG |
| avrBs2-R544A-F | CGCCTATTCGGTGAGCTACGCGTTTGAGGACTTTTCCGTGC |
| avrBs2-R544A-R | GCACGGAAAAGTCCTCAAACGCGTAGCTCACCGAATAGGCG |
| avrBs2-D547A-F | GTGAGCTACCGGTTTGAGGCCTTTTCCGTGCCGCGCGCC |
| avrBs2-D547A-R | GGCGCGCGGCACGGAAAAGGCCTCAAACCGGTAGCTCAC |
| avrBs2-D596A-F | GGCGAAAGCCTGCTGACCGCCCAGGCCGAGGCGGAATTG |
| avrBs2-D596A-R | CAATTCCGCCTCGGCCTGGGCGGTCAGCAGGCTTTCGCC |
| pVSP61-avrBS2-BamHI-F | AAAGGATCCGCCATTGTCGCTGGCAG |
| pVSP61-avrBs2-HA-SalI-R | AAAGTCGACTCAAGCGTAGTCTGGGACGTCGTATGGGTACTCCGGCTCGGTCTGGTTGGC |
| pGD-avrBs2-F | ATCACCAGTCTCTCTCTACAAGATCTATGCGTATAGGTCCTCCGCA |
| pGD-avrBs2-Flag-R | ATCAGTTATCTAGATCCGGTGGATCCTCACTTATCGTCGTCATCCTTGTAATCCTCCGGCTCGGTCTGGTTGG |
| pGD-avrBs2-GFP-R | TGCAGAATTCGAAGCTTGCTCCGGCTCGGTCTGGTTGGC |
| pGD-avrBs2-Flag-R | ATCAGTTATCTAGATCCGGTGGATCCTCACTTATCGTCGTCATCCTTGTAATCCTCCGGCTCGGTCTGGTTGG |
| pGEX4T-3-avrBs2-BamHI-F | AAAggatccATGCGTATAGGTCCTCCGCA |
| pGEX4T-3-avrBs2-XhoI-R | AAACTCGAGTCACTCCGGCTCGGTCTGGTTGG |
| pColdSUMO-avrBs2-F | GGCTCACCGCGAACAGATTGGAGGCATGCGTATAGGTCCTCCGCA |
| pColdSUMO-avrBs2-R | ACAAGCTTGAATTCGGATCCCTCGAGTCACTCCGGCTCGGTCTGGT |
| pET28a-cGFP-F | AAGCTTGCGGCCGCACTCGAGATGGTGAGCAAGGGCGAGG |
| pET28a-cGFP-R | GTGGTGGTGGTGGTGTTACTTGTACAGCTCGTCCAT |
| pET28a-avrBs2-GFP-F | CAGCAAATGGGTCGCGGATCCATGCGTATAGGTCCTCCG |
| pET28a-avrBs2-GFP-R | TGCGGCCGCAAGCTTGTCGACCTCCGGCTCGGTCTG |
| avrBs2-Xe37-del-F | TAAAACGACGGCCAGTGCCAAGCTTTGGTGGAGGCACTGCTAA |
| avrBs2-Xe37-del-mid-R | CGAAGCCGTGATTGGAAGGTATGACAATTGGCAAATTAGG |
| avrBs2-Xe37-del-mid-F | CCTAATTTGCCAATTGTCATACCTTCCAATCACGGCTTCG |
| avrBs2-Xe37-del-R | ATGACCATGATTACGAATTCGAGCTCATTGATGATGTCCACGATGG |
| avrBs2-Xcitri-del-F | TAAAACGACGGCCAGTGCCAAGCTTGGACAACGACGCCCATTG |
| avrBs2-Xcitri-del-mid-R | GTTGGAAGGCGATGACCTCGAAAACGCGGC |
| avrBs2-Xcitri-del-mid-F | CGAGGTCATCGCCTTCCAACCTCACGGCTT |
| avrBs2-Xcitri-del-R | ATGACCATGATTACGAATTCGAGCTCATCTGTTCCGCACCTGGC |
| pVSP61-avrBs2-Xe37-F | GACGGCCAGTGAATTCCCGGGGATCCTGGTGGAGGCACTGCTAA |
| pVSP61-avrBs2-Xe37-R | TACGCCAAGCTTGGCTGCAGGTCGACTCAAGCGTAGTCTGGGACGTCGTATGGGTAATCCGTCTCCGTCTGCCTGG |
| pVSP61-avrBs2-Xcitri-F | GACGGCCAGTGAATTCCCGGGGATCCGGACAACGACGCCCATTG |
| pVSP61-avrBs2-Xcitri-R | TACGCCAAGCTTGGCTGCAGGTCGACTCAAGCGTAGTCTGGGACGTCGTATGGGTAATCCGTCTCCGTCTGCTTGG |
| pK18msB-hrpF-del-F | ACAGCTATGACATGATTACGAATTCCGGTATTCGCTGCTGTTT |
| hrpF-del-mid-R | ACCTCGCTGTGGTATGCGCGATAGGCGGCCTCGCGAATGA |
| hrpF-del-mid-F | TCATTCGCGAGGCCGCCTATCGCGCATACCACAGCGAGGT |
| pK18msB-hrpF-del-R | GCCTGCAGGTCGACTCTAGAGGATCCAAGGGTGCTGAGATTGGA |
| pUFR80-xanT-BamHI-F | AAAGGATCCCATCGTTGTCCATCCAGA |
| pUFR80-XlyR-BamHI-F | aaaGGATCCACCCAGAAACGCAAACTC |
| XlyR-del-mid-R | ATGACGCATGGCAGGGCAAAACGTCCGTCG |
| XlyR-del-mid-F | TTTGCCCTGCCATGCGTCATGCACGTTGAC |
| pUFR80-XlyR-KpnI-R | AAAGGTACCAGATTGAAGACGATGCGAC |
| xanT-del-mid-R | ACGACGACATGGGAAAGCCCCGCATGCGGATGCGGTGGGC |
| xanT-del-mid-F | GCCCACCGCATCCGCATGCGGGGCTTTCCCATGTCGTCGT |
| pUFR80-xanT-KpnI-R | aaaGGTACCCGATACGCCGTTTGTTCT |
| xanP-del-HindIII-F | TTTAAGCTTaactgagcggaactgtctg |
| xanP-del-mid-R | GTCGCCGCCTCCACTCGAGCGGGAAAGCCCTCAGAGTTTG |
| xanP-del-mid-F | caaactctgagggctttcccgctcgagtggaggcggcgac |
| xanP-del-BamHI-R | TTTGGATCCGCTGTCTGGCTGTCATT |
| xanT&xanP-del-HindIII-F | TTTAAGCTTCATCGTTGTCCATCCAGA |
| xanT&xanP-del-mid-R | GTCGCCGCCTCCACTCGAGCCGCATGCGGATGCGGTGGGC |
| xanT&xanP-del-mid-F | GCCCACCGCATCCGCATGCGgctcgagtggaggcggcgac |
| xanP-28a-NdeI-F | AAAGCATATGatgtcgtcgtttctggacac |
| xanP-28a-EcoRI-R | aaaGAATTCTCAGACCTCATGCATCGTGC |
| xanP-H99A,T101A-mid-F | tgctgccgccgagcagctacgccgtggccttattgggcggggtcaatga |
| xanP-H99A,T101A-mid-R | TCATTGACCCCGCCCAATAAGGCCACGGCGTAGCTGCTCGGCGGCAGCA |
| xanP-H204A,S206A-F | actacatcaacttccagtttgctattgcgctggcctacctgtgcgacac |
| xanP-H204A,S206A-R | GTGTCGCACAGGTAGGCCAGCGCAATAGCAAACTGGAAGTTGATGTAGT |
| pC1305-xanP-KpnI-F | AAAGGTACCatgtcgtcgtttctggacac |
| pC1305-xanP-HindIII-R | TTTAAGCTTGACCTCATGCATCGTGCGAA |
| pUFR80-gC-TP-BamHI-F | aaaGGATCCAGCGACAATGGCGGATT |
| pUFR80-gC-TP-HindIII-R | TTTAAGCTTATCGGGTAGTGGTGTTGG |
| xanT-Del366-F | CCACGCTATGTGACGGTGCGGGCCTCAACGGCAACTACAA |
| xanT-Del366-R | TTGTAGTTGCCGTTGAGGCCCGCACCGTCACATAGCGTGG |
| xanT-pUFR80-HindIII-R | TTTAAGCTTCGATACGCCGTTTGTTCT |
| xanTP-polycistron check-F | AGGGCTACGAATACCGCAT |
| xanTP-polycistron check-R | TGACCGACGAAGGTATTGC |
| pBBR-pPilA-xanTP-HR-F | CTGCTTCCGGTAGTCAATAAACCGGTGTCAAACTGAACGGGATGTT |
| pBBR-pPilA-xanTP-mid-R | TGGGGATGCAACAAGGGCATGGATGCATTCCCTAGAGGGT |
| pBBR-pPilA-xanTP-mid-F | ACCCTCTAGGGAATGCATCCATGCCCTTGTTGCATCCCCA |
| pBBR-pPilA-xanTP-HR-R | AATGAATTACAACAGTTTTTATGCATTCAGACCTCATGCATCGTGC |
| avrBs2-qPCR-F | CTTACGCGAAGGGCTATCGT |
| avrBs2-qPCR-R | AACGGCATTTCACGCAACTC |
| Xoc-16S-qPCR-F | TATCTAATCCTGTTTGCTCCCC |
| Xoc-16S-qPCR-R | AGAGTGTGGTAGAGGGTAGC |
| xanT-qPCR-F | GAGCACGGTCGAGTATCAGG |
| xanT-qPCR-R | TTACTGGCAAACGCAAAGCC |
| xanP-qPCR-F | GCGCTAACGACAATCTGCTG |
| xanP-qPCR-R | GATACGCCGTTTGTTCTGCC |
| OsActin1-qPCR-F | AGTCTGGCCCATCCATTGTG |
| OsActin1-qPCR-R | AGAAACAAGCAGGAGGACGG |
| pVSP61-xanP-F | GACGGCCAGTGAATTCCCGGGGATCCATGTCGTCGTTTCTGGACAC |
| pVSP61-xanP-3FLAG-R | GCTATGACCATGATTACGCCAAGCTTCACTTATCGTCATCGTCCTTGTAATCGATGTCGTGATCCTTATAGTCTCCATCATGGTCTTTGTAGTCGACCTCATGCATCGTGCGAA |
